# Supplementary material for: A robust model for cell type-specific interindividual variation in single-cell RNA sequencing data
Source: Nat Commun. 2024 Jun 19;15:5229. doi: 10.1038/s41467-024-49242-9 (PMC11186839; doi:10.1038/s41467-024-49242-9)
Supplement: Supplementary file 1 — Supplementary Information [file 41467_2024_49242_MOESM1_ESM.pdf]

Supplementary Note 1

1 Modelling Overall Pseudobulk data

We start with the model for Overall Pseudobulk (OP) data, which sums expression over all measured cells for each individual. The model in Eq 5 in the main text can be written as

$$\mathbf{y} = \mathbf{P}\boldsymbol{\beta} + \boldsymbol{\alpha} + (\mathbf{I} \bullet \mathbf{P})\text{vec}\left(\boldsymbol{\Gamma}^T\right) + \boldsymbol{\delta}$$

Here, $\mathbf{y} \in \mathbb{R}^{N\times 1}$ is the vector of overall pseudobulk expression, where N is the number of donor individuals. $\mathbf{P} \in \mathbb{R}^{N\times C}$ is the matrix of cell type proportions, where C is the number of cell types. (Note that C is assumed known and fixed, as is the assignment of cells to cell types.) $\boldsymbol{\beta} \in \mathbb{R}^{C\times 1}$ is the vector of cell type fixed effects, which represent the mean expression level for each cell type. $\boldsymbol{\alpha} \in R^{N\times 1}$ is the vector of average expression across individuals that is homogeneously shared across all cell types, which we treat as a random effect:

$$\boldsymbol{\alpha} \sim \mathcal{N}\left(0, \sigma_\alpha^2 I\right)$$

where $\mathbf{I}$ is the identity matrix.

$\boldsymbol{\Gamma} \in \mathbb{R}^{N\times C}$ is the matrix of cell type-specific average expression across all individuals and cell types, which we also treat as a random effect:

$$\boldsymbol{\Gamma} \sim \mathcal{MN}\left(0, \mathbf{I}, \mathbf{V}\right)$$

This is written as a matrix normal random variable, where $\mathbf{I}$ is the row-covariance matrix and $\mathbf{V}$ is the column-covariance matrix. (Equivalently, we assume that $\text{vec}\left(\boldsymbol{\Gamma}^T\right) \sim \mathcal{N}\left(0, \mathbf{I} \otimes \mathbf{V}\right)$, where $\text{vec}\left(\right)$ concatenates columns of a matrix into a vector.) In words, this means that we assume $\boldsymbol{\Gamma}$ is i.i.d. across individuals and has covariance $\mathbf{V}$ across cell types.

$\bullet$ is the transposed Khatri-Rao product. Although it may seem complex, $(\mathbf{A} \bullet \mathbf{B})$ is simply Kronecker product of corresponding rows of $\mathbf{A}$ and $\mathbf{B}$. This generalizes the standard interaction between two univariate features to two feature matrices, and is implicitly constructed in any linear regression involving interactions.

Finally, $\boldsymbol{\delta} \in \mathbb{R}^{N\times 1}$ is the vector of measurement noise due to sampling a finite number of cells. We assume that $\boldsymbol{\delta} \sim \mathcal{N}\left(0, D\right)$, where $\mathbf{D} := \text{diag}\left(\boldsymbol{\nu}\right)$ and $\boldsymbol{\nu} \in R^{N\times 1}$ is the vector of noise levels for overall pseudobulk noise. Equivalently, this can be written $\boldsymbol{\delta}_i \overset{\text{ind}}{\sim} \mathcal{N}\left(0, \boldsymbol{\nu}_i\right)$. In our derivations, we assume that $\boldsymbol{\nu}$ is known. In practice, we calculate it by evaluating the variance across cells (see main text for details), but we explore violations of this assumption in simulations (see main text and Section 3.3 below).

In practice, it is often essential to include other covariates in the model as either fixed or random effects, such as experiment batch, age, and sex. For simplicity and statistical parsimony, we assume

that the effects of these covariates are shared homogeneously across cell types. We can include these effects by slightly generalizing our above model:

$$\mathbf{y} = \mathbf{Xb} + \boldsymbol{\alpha} + (\mathbf{I} \bullet \mathbf{P})\text{vec}\left(\boldsymbol{\Gamma}^T\right) + \mathbf{Z}\boldsymbol{\mu} + \boldsymbol{\delta}$$

where $\mathbf{b}$ is the vector of fixed effects with the design matrix $\mathbf{X}$, which now includes both traditional covariates (e.g., age and sex) as well as the cell type fixed effects $\boldsymbol{\beta}$, and $\boldsymbol{\mu}$ is the vector of random effects with the design matrix $\mathbf{Z}$, such as batch effects. It can be useful to partition $\mathbf{Z}\boldsymbol{\mu}$ as:

$$\mathbf{Z}\boldsymbol{\mu} = \begin{bmatrix}\mathbf{Z_1} & ... & \mathbf{Z_r}\end{bmatrix} \begin{bmatrix}\boldsymbol{\mu_1} \\ ... \\ \boldsymbol{\mu_r}\end{bmatrix} = \sum_{i=1}^{r} \mathbf{Z_i}\boldsymbol{\mu_i}$$

where $\mathbf{Z_i}$ and $\boldsymbol{\mu_i}$ are the design matrix and the random effect vector for feature i. We assume that all random effects are distributed $\boldsymbol{\mu_i} \overset{\text{ind}}{\sim} \mathcal{N}\left(0, \sigma_i^2\mathbf{I}\right)$.

Let $\circ$ be the element-wise (or Hadamard) product, and let $\otimes$ be the Kronecker (or tensor) product. Under this more general model of OP data that includes random effects of a factor variable $\mathbf{Z}$, the distribution of $\mathbf{y}$ is:

$$\begin{aligned}
\mathbb{E}\left(\mathbf{y}\right) =& \mathbf{Xb} \\
\mathbb{V}\left(\mathbf{y}\right) =& \mathbb{V}\left(\boldsymbol{\alpha}\right) + \mathbb{V}\left((\mathbf{I} \bullet \mathbf{P})\text{vec}\left(\boldsymbol{\Gamma}^T\right)\right) + \sum \mathbb{V}\left(\mathbf{Z_i}\boldsymbol{\mu_i}\right) + \mathbb{V}\left(\boldsymbol{\delta}\right) \\
=& \sigma_\alpha^2\mathbf{I} + (\mathbf{I} \bullet \mathbf{P})\mathbb{V}\left(\text{vec}\left(\boldsymbol{\Gamma}^T\right)\right)(\mathbf{I} \bullet \mathbf{P})^T + \sum \mathbf{Z_i}\mathbb{V}\left(\boldsymbol{\mu_i}\right)\mathbf{Z_i}^T + \mathbf{D} \\
=& \sigma_\alpha^2\mathbf{I} + (\mathbf{I} \bullet \mathbf{P})(\mathbf{I} \otimes \mathbf{V})(\mathbf{I} \bullet \mathbf{P})^T + \sum \mathbf{Z_i}\sigma_i^2\mathbf{Z_i}^T + \mathbf{D} \\
=& \sigma_\alpha^2\mathbf{I} + \mathbf{I} \circ (\mathbf{PVP}^T) + \sum_{i=1}^{r} \sigma_i^2\mathbf{Z_i}\mathbf{Z_i}^T + \mathbf{D} \\
\implies \mathbf{y} \sim& \mathcal{N}\left(\mathbf{Xb}, \sigma_\alpha^2\mathbf{I} + \mathbf{I} \circ (\mathbf{PVP}^T) + \sum_{i=1}^{r} \sigma_i^2\mathbf{Z_i}\mathbf{Z_i}^T + \mathbf{D}\right) \qquad (1)
\end{aligned}$$

1.1 Fitting Overall Pseudobulk with ML, REML, and HE

ML

We fit maximum likelihood (ML) estimates of $\boldsymbol{\beta}$, σ_α^2, and $\mathbf{V}$ by maximizing the log-likelihood function jointly over these parameters (as well as over the other fixed effects in $\mathbf{b}$ and the random effect variance components σ_i^2):

$$l(\boldsymbol{b}, \sigma_\alpha^2, \mathbf{V}, \sigma_1^2, ..., \sigma_r^2 | \mathbf{y}) = -\frac{1}{2}[const + \ln\left|\mathbb{V}\left(\mathbf{y}\right)\right| + (\mathbf{y} - \mathbf{Xb})^T(\mathbb{V}\left(\mathbf{y}\right))^{-1}(\mathbf{y} - \mathbf{Xb})]$$

where *const* is a constant and $\mathbb{V}\left(\mathbf{y}\right)$ means the covariance matrix of the vector $\mathbf{y}$ that depends on σ_α^2 and $\mathbf{V}$ (and σ_i^2), as shown in Eq 1.

REML

For restricted maximum likelihood (REML) estimates of $\mathbf{V}$ (and σ_i^2), we instead maximize the restricted likelihood (Harville, 1974; Harville, 1977):

$$l(\sigma_\alpha^2, \mathbf{V}, \sigma_1^2, ..., \sigma_r^2 | y) = -\frac{1}{2}[const + \ln|\mathbb{V}(\mathbf{y})| + \ln|\mathbf{X}^T \mathbb{V}(\mathbf{y})^{-1} \mathbf{X}| + \mathbf{y}^T \mathbf{H} \mathbf{y}]$$

where $\mathbf{H}$ is defined as:

$$\mathbf{H} := \mathbb{V}(\mathbf{y})^{-1} - \mathbb{V}(\mathbf{y})^{-1} \mathbf{X}(\mathbf{X}^T \mathbb{V}(\mathbf{y})^{-1} \mathbf{X})^{-1} \mathbf{X}^T \mathbb{V}(\mathbf{y})^{-1}$$

After fitting these variance components, we then fit the fixed effects in REML using generalized least squares.

For both ML and RMEL, we maximize the likelihood function using the BFGS algorithm implemented in the R function 'optim'. To initialize optimization, in ML, we initialize fixed effects with ordinary least square estimates and initialize variances of random effects with parameters such that each variance component explains an equal amount of residual OP variance after subtracting off the fixed effects; in REML, since fixed effect sizes are not involved in the likelihood function, we initialize variances of random effects with parameters such that each variance component explains an equal amount of OP variance. If the initial optimization attempt fails to converge or has one variance component that explains more than 5 folds of OP variance, we rerun optimization for 10 times with random initial parameters and pick the converged run with the largest likelihood as the final result, in order to mitigate bias from local maxima. We allow negative variance components to reduce bias, though the total expression variance is always positive.

HE

For Haseman-Elston regression (HE), we estimate variance components by method-of-moments after residualizing out fixed effects. This residualization uses the projection matrix $\mathbf{M} := \mathbf{I} - \mathbf{X}(\mathbf{X}^T\mathbf{X})^{-1}\mathbf{X}^T$:

$$\mathbf{y}' := \mathbf{M}\mathbf{y} = \mathbf{M}[\boldsymbol{\alpha} + (\mathbf{I} \bullet \mathbf{P})\text{vec}\left(\boldsymbol{\Gamma}^T\right) + \mathbf{Z}\boldsymbol{\mu} + \boldsymbol{\delta}]$$

Note that $\mathbf{M}$ is symmetric ($\mathbf{M}^T = \mathbf{M}$) and idempotent ($\mathbf{M}^2 = \mathbf{M}$).

The HE estimator of $\boldsymbol{\theta} := (\sigma_\alpha^2, \mathbf{V}_{11}, ..., \mathbf{V}_{CC}, \mathbf{V}_{12}, ..., \mathbf{V}_{(C-1)(C)}, \sigma_1^2, ..., \sigma_r^2)$ is obtained by minimizing the squared error between the expected and observed sample covariance matrix, i.e., the standard method-of-moments:

$$\begin{aligned}
\widehat{\boldsymbol{\theta}} &= argmin_{\boldsymbol{\theta}} ||\mathbf{y}'\mathbf{y}'^T - \mathbb{V}(\mathbf{y}')||_F \\
&= argmin_{\boldsymbol{\theta}} ||\mathbf{M}\mathbf{y}\mathbf{y}^T\mathbf{M}^T - \mathbf{M}\mathbb{V}(\mathbf{y})\mathbf{M}||_F \\
&= argmin_{\boldsymbol{\theta}} ||\mathbf{M}\mathbf{y}\mathbf{y}^T\mathbf{M} - \mathbf{M}[\sigma_\alpha^2\mathbf{I} + \mathbf{I} \circ (\mathbf{P}\mathbf{V}\mathbf{P}^T) + \sum_{i=1}^{r} \sigma_i^2 \mathbf{Z_i}\mathbf{Z_i}^T + \mathbf{D}]\mathbf{M}||_F \\
&= argmin_{\boldsymbol{\theta}} ||(\mathbf{M}\mathbf{y}\mathbf{y}^T\mathbf{M} - \mathbf{M}\mathbf{D}\mathbf{M}) - \mathbf{M}[\sigma_\alpha^2\mathbf{I} + \mathbf{I} \circ (\mathbf{P}\mathbf{V}\mathbf{P}^T) + \sum_{i=1}^{r} \sigma_i^2 \mathbf{Z_i}\mathbf{Z_i}^T]\mathbf{M}||_F
\end{aligned}$$

To solve this, rewrite it as a linear function of $\boldsymbol{\theta}$,

$$\begin{aligned}
&\mathrm{vec}\left(\mathbf{M}[\sigma_\alpha^2\mathbf{I}+\mathbf{I}\circ(\mathbf{PVP}^T)+\sum_{i=1}^r\sigma_i^2\mathbf{Z_i}\mathbf{Z_i}^T]\mathbf{M}\right)\\
&=\mathrm{vec}\left(\mathbf{MIM}\right)\sigma_\alpha^2+\mathrm{vec}\left(\mathbf{M}[\mathbf{I}\circ(\mathbf{PVP}^T)]\mathbf{M}\right)+\sum_{i=1}^r\mathrm{vec}\left(\mathbf{MZ_iZ_i}^T\mathbf{M}\right)\sigma_i^2\\
&=\mathrm{vec}\left(\mathbf{M}\right)\sigma_\alpha^2+\mathrm{vec}\left(\mathbf{M}[\mathbf{I}\circ(\sum_{m,n}\mathbf{V}_{mn}\mathbf{P}_{,m}(\mathbf{P}_{,n})^T)]\mathbf{M}\right)+\sum_{i=1}^r\mathrm{vec}\left(\mathbf{MZ_iZ_i}^TM\right)\sigma_i^2\\
&=\mathrm{vec}\left(\mathbf{M}\right)\sigma_\alpha^2+\mathrm{vec}\left(\sum_{m,n}\mathbf{M}[\mathbf{I}\circ(\mathbf{P}_{,m}(\mathbf{P}_{,n})^T)]\mathbf{M}\mathbf{V}_{mn}\right)+\sum_{i=1}^r\mathrm{vec}\left(\mathbf{MZ_iZ_i}^T\mathbf{M}\right)\sigma_i^2\\
&=\mathrm{vec}\left(\mathbf{M}\right)\sigma_\alpha^2+\sum_{m,n}\mathrm{vec}\left(\mathbf{M}[\mathbf{I}\circ(\mathbf{P}_{,m}(\mathbf{P}_{,n})^T)]\mathbf{M}\right)\mathbf{V}_{mn}+\sum_{i=1}^r\mathrm{vec}\left(\mathbf{MZ_iZ_i}^T\mathbf{M}\right)\sigma_i^2\\
&=\mathbf{Q}\theta
\end{aligned}$$

Now we use the standard OLS (ordinary least square) projection of $\mathrm{vec}\left(\mathbf{Myy}^T\mathbf{M}-\mathbf{MDM}\right)$ onto the span of $\mathbf{Q}$ to estimate $\boldsymbol{\theta}$, with $\widehat{\boldsymbol{\theta}}=(\mathbf{Q}^T\mathbf{Q})^{-1}\mathbf{Q}^T\mathrm{vec}\left(\mathbf{Myy}^T\mathbf{M}-\mathbf{MDM}\right)$.

Here, $\mathbf{Q}^T:=\begin{bmatrix}\mathrm{vec}\left(\mathbf{M}\right)^T\\ \mathrm{vec}\left(\mathbf{M}[\mathbf{I}\circ(\mathbf{P}_{,1}(\mathbf{P}_{,1})^T)]\mathbf{M}\right)^T\\ \ldots\\ \mathrm{vec}\left(\mathbf{M}[\mathbf{I}\circ(\mathbf{P}_{,C}(\mathbf{P}_{,C})^T)]\mathbf{M}\right)^T\\ 2\mathrm{vec}\left(\mathbf{M}[\mathbf{I}\circ(\mathbf{P}_{,1}(\mathbf{P}_{,2})^T)]\mathbf{M}\right)^T\\ \ldots\\ 2\mathrm{vec}\left(\mathbf{M}[\mathbf{I}\circ(\mathbf{P}_{,C-1}(\mathbf{P}_{,C})^T)]\mathbf{M}\right)^T\\ \mathrm{vec}\left(\mathbf{MZ_1Z_1}^T\mathbf{M}\right)^T\\ \ldots\\ \mathrm{vec}\left(\mathbf{MZ_rZ_r}^T\mathbf{M}\right)^T\end{bmatrix}$. There is a factor of 2 because $\mathbf{V}$ is symmetric.

In special cases, $\mathbf{Q}$ can be simplified. In Hom model where $\mathbf{V}=0$, we have $\boldsymbol{\theta}=(\sigma_\alpha^2,\sigma_1^2,...,\sigma_r^2)$ and $\mathbf{Q}^T=\begin{bmatrix}\mathrm{vec}\left(\mathbf{M}\right)^T\\ \mathrm{vec}\left(\mathbf{MZ_1Z_1}^T\mathbf{M}\right)^T\\ \ldots\\ \mathrm{vec}\left(\mathbf{MZ_rZ_r}^T\mathbf{M}\right)^T\end{bmatrix}$; in Free model where $\mathbf{V}_{m,n}=0$ when $m\neq n$, we have $\boldsymbol{\theta}=$

$(\sigma_\alpha^2, \mathbf{V}_{11}, ..., \mathbf{V}_{CC}, \sigma_1^2, ..., \sigma_r^2)$ and $\mathbf{Q}^T = \begin{bmatrix} \text{vec}\,(\mathbf{M})^T \\ \text{vec}\left(\mathbf{M}[\mathbf{I} \circ (\mathbf{P}_{,1}(\mathbf{P}_{,1})^T)]\mathbf{M}\right)^T \\ ... \\ \text{vec}\left(\mathbf{M}[\mathbf{I} \circ (\mathbf{P}_{,C}(\mathbf{P}_{,C})^T)]\mathbf{M}\right)^T \\ \text{vec}\left(\mathbf{M}\mathbf{Z_1}\mathbf{Z_1}^T\mathbf{M}\right)^T \\ ... \\ \text{vec}\left(\mathbf{M}\mathbf{Z_r}\mathbf{Z_r}^T\mathbf{M}\right)^T \end{bmatrix}$

Note that this expression is far more efficient than naively solving the linear system of equations directly because it takes advantage of the implicit structure in the $\mathbf{Q}$ matrix.

2 Modelling Cell Type-specific Pseudobulk data

The Cell Type-specific Pseudobulk (CTP) data uses the same model on single cells as the OP data. However, in CTP, each individual's scRNA data is collapsed into a vector where each entry sums over all cells in a given cell type, rather than a single number that sums over all cells in all cell types. Formally, the Eq 6 model in the main text can be written as:

$$\mathbf{Y} = \mathbf{1}_N\boldsymbol{\beta}^T + \boldsymbol{\alpha}\mathbf{1_C}^T + \boldsymbol{\Gamma} + \boldsymbol{\delta}$$

As in the OP model, N is the number of individuals, C is the number of cell types, $\boldsymbol{\beta}$ is the mean expression in each cell type (shared across individuals), $\boldsymbol{\alpha}$ is the mean expression in each individual (shared across cell types), and $\boldsymbol{\Gamma}$ are cell type-specific variations across individuals. $\mathbf{1_N}$ is a vector of 1s with length N, and $\mathbf{1_C}$ is a vector of 1s with length C. We assume the same random effect distributions on $\boldsymbol{\alpha}$ and $\boldsymbol{\Gamma}$ as in the OP data.

The CTP data is a matrix $\mathbf{Y} \in R^{N \times C}$. The OP data is roughly equal to the average across columns of the CTP data, and this holds exactly in the special case where all cell types are uniformly sampled in all individuals.

$\boldsymbol{\delta}$ is now slightly different than in the OP data, as it is an $N \times C$ matrix instead of an $N \times 1$ vector. The difference is analogous to the difference between $\mathbf{y}$ in the OP data and $\mathbf{Y}$: approximately, the OP $\boldsymbol{\delta}$ can be thought of as averaging across the columns of the CTP data. Its distribution is given by $\boldsymbol{\delta}_{ic} \overset{\text{ind}}{\sim} \mathcal{N}(0, \boldsymbol{\nu}_{ic})$, where $\boldsymbol{\nu}_{ic}$ is analogous to the $\boldsymbol{\nu}_i$ above for OP data.

As in the OP above, we include extra covariates as either fixed or random effects, to represent other factors that are shared across cell types, such as experiment batch, age, and sex.

$$\mathbf{Y} = \mathbf{A}\boldsymbol{\zeta}\mathbf{1_C}^T + \mathbf{1_N}\boldsymbol{\beta}^T + \boldsymbol{\alpha}\mathbf{1_C}^T + \boldsymbol{\Gamma} + \mathbf{Z}\boldsymbol{\mu}\mathbf{1_C}^T + \boldsymbol{\delta}$$

Here, $\boldsymbol{\zeta}$ is the vector of fixed effects (except for cell type fixed effect), $\mathbf{A}$ is the corresponding deign matrix. There is a vector $\mathbf{1}_C^T$ because there are C cell types per individual, and the effect is assumed to be shared across cell types. The same as in the OP, $\boldsymbol{\mu}$ is the vector of random effects, except for overall random effect and cell type-specific random effect, $\mathbf{Z}$ is the design matrix for random effects.

We define $\mathbf{y} := \text{vec}\left(\mathbf{Y}^T\right)$ for this section, but note that this is not the same as the OP data in $\mathbf{y}$ in Section 1.

$$
\begin{aligned}
\mathbf{y} =&\text{vec}\left(\mathbf{1_C}\boldsymbol{\zeta}^T\mathbf{A}^T\right) + \text{vec}\left(\boldsymbol{\beta}\mathbf{1_N}^T\right) + \text{vec}\left(\mathbf{1_C}\boldsymbol{\alpha}^T\right) + \text{vec}\left(\boldsymbol{\Gamma}^T\right) + \text{vec}\left(\mathbf{1_C}\boldsymbol{\mu}^T\mathbf{Z}^T\right) + \text{vec}\left(\boldsymbol{\delta}^T\right) \\
=&(\mathbf{A}\otimes\mathbf{1_C})\boldsymbol{\zeta} + (\mathbf{1_N}\otimes\mathbf{I_C})\boldsymbol{\beta} + (\mathbf{I_N}\otimes\mathbf{1_C})\boldsymbol{\alpha} + \text{vec}\left(\boldsymbol{\Gamma}^T\right) + (\mathbf{Z}\otimes\mathbf{1_C})\boldsymbol{\mu} + \text{vec}\left(\boldsymbol{\delta}^T\right) \\
=&\mathbf{Xb} + (\mathbf{I_N}\otimes\mathbf{1_C})\boldsymbol{\alpha} + \text{vec}\left(\boldsymbol{\Gamma}^T\right) + \sum_{i=1}^{r}(\mathbf{Z_i}\otimes\mathbf{1_C})\boldsymbol{\mu}_i + \text{vec}\left(\boldsymbol{\delta}^T\right)
\end{aligned}
$$

with $\mathbf{X} := \begin{bmatrix}\mathbf{A}\otimes\mathbf{1_C} & \mathbf{1_N}\otimes\mathbf{I_C}\end{bmatrix}$ and $\mathbf{b} := \begin{bmatrix}\boldsymbol{\zeta} \\ \boldsymbol{\beta}\end{bmatrix}$.

The expectation and variance of $\mathbf{Y}$ is:

$$
\begin{aligned}
\mathbb{E}(\mathbf{y}) =&\mathbf{Xb} \\
\mathbb{V}(\mathbf{y}) =&\mathbb{V}\left((\mathbf{I_N}\otimes\mathbf{1_C})\boldsymbol{\alpha}\right) + \mathbb{V}\left(\text{vec}\left(\boldsymbol{\Gamma}^T\right)\right) + \sum_{i=1}^{r}\mathbb{V}\left((\mathbf{Z_i}\otimes\mathbf{1_C})\boldsymbol{\mu}_i\right) + \mathbb{V}\left(\text{vec}\left(\boldsymbol{\delta}^T\right)\right) \\
=&(\mathbf{I_N}\otimes\mathbf{1_C})(\sigma_\alpha^2\mathbf{I_N})(\mathbf{I_N}\otimes\mathbf{1_C})^T + \mathbf{I_N}\otimes\mathbf{V} + \sum_{i=1}^{r}(\mathbf{Z_i}\otimes\mathbf{1_C})\sigma_i^2(\mathbf{Z_i}\otimes\mathbf{1_C})^T + \mathbf{D} \\
=&(\mathbf{I_N}\mathbf{I_N})\otimes(\mathbf{1_C}\mathbf{1_C}^T)\sigma_\alpha^2 + \mathbf{I_N}\otimes\mathbf{V} + \sum_{i=1}^{r}(\mathbf{Z_i}\mathbf{Z_i}^T)\otimes(\mathbf{1_C}\mathbf{1_C}^T)\sigma_i^2 + \mathbf{D} \\
=&(\mathbf{I_N}\otimes\mathbf{J_C})\sigma_\alpha^2 + \mathbf{I_N}\otimes\mathbf{V} + \sum_{i=1}^{r}[(\mathbf{Z_i}\mathbf{Z_i}^T)\otimes\mathbf{J_C}]\sigma_i^2 + \mathbf{D} \\
=&\mathbf{I_N}\otimes(\mathbf{J_C}\sigma_\alpha^2 + \mathbf{V}) + \sum_{i=1}^{r}[(\mathbf{Z_i}\mathbf{Z_i}^T)\otimes\mathbf{J_C}]\sigma_i^2 + \mathbf{D} \\
&\implies \\
&\mathbf{y}\sim\mathcal{N}\left(\mathbf{Xb}, \mathbf{I_N}\otimes(\mathbf{J_C}\sigma_\alpha^2 + \mathbf{V}) + \sum_{i=1}^{r}[(\mathbf{Z_i}\mathbf{Z_i}^T)\otimes\mathbf{J_C}]\sigma_i^2 + \mathbf{D}\right) \qquad (2)
\end{aligned}
$$

where $\mathbf{J_C}$ is a $C\times C$ matrix of 1s.

2.1 Fitting Cell Type-specific Pseudobulk with ML, REML, and HE

ML and REML

These approaches are identical to the OP section, except that the CTP likelihood (or restricted likelihood) from (2) is used in place of the OP likelihood from (1).

For both ML and REML, we maximize the likelihood function using the BFGS algorithm implemented in the R function ‘optim’. To initialize optimization, in ML, we initialize fixed effects with ordinary least square estimates and initialize variances of random effects with parameters such that each variance component explains an equal amount of residual CTP variance after subtracting off the fixed effects; in REML, since fixed effect sizes are not involved in the likelihood function, we initialize variances of random effects with parameters such that each variance component explains an equal amount of CTP variance. If the initial optimization attempt fails to converge, or has one

variance component that explains more than 5 times OP variance, or has estimates equal to the initial values, we rerun optimization 10 times with random initial parameters and pick the converged run with the largest likelihood as the final result, in order to mitigate bias from local maxima. We allow negative variance components to reduce bias, though the total expression variance is always positive.

HE

As for OP data, we first projected out fixed effect by $\mathbf{M} := \mathbf{I} - \mathbf{X}(\mathbf{X}^T\mathbf{X})^{-1}\mathbf{X}^T$:

$$\mathbf{y'} := \mathbf{My} = \mathbf{M}[(\mathbf{I_N} \otimes \mathbf{1_C})\alpha + \text{vec}\left(\mathbf{\Gamma}^T\right) + \sum_{i=1}^{r}(\mathbf{Z_i} \otimes \mathbf{1_C})\boldsymbol{\mu_i} + \text{vec}\left(\boldsymbol{\delta}^T\right)]$$

The HE estimator of $\boldsymbol{\theta} := (\sigma_\alpha^2, \mathbf{V}_{11}, ..., \mathbf{V}_{CC}, \mathbf{V}_{12}, ..., \mathbf{V}_{(C-1)(C)}, \sigma_1^2, ..., \sigma_r^2)$ is obtained by minimizing the squared error between the expected and observed sample covariance matrix:

$$\begin{aligned}
\widehat{\boldsymbol{\theta}} =& argmin_{\boldsymbol{\theta}} ||\mathbf{y'}\mathbf{y'}^T - \mathbb{V}\left(y\right)'||_F \\
=& argmin_{\boldsymbol{\theta}} ||\mathbf{M}\mathbf{y}\mathbf{y}^T\mathbf{M}^T - \mathbf{M}\mathbb{V}\left(\mathbf{y}\right)\mathbf{M}||_F \\
=& argmin_{\boldsymbol{\theta}} ||\mathbf{M}\mathbf{y}\mathbf{y}^T\mathbf{M} - \mathbf{M}[\mathbf{I_N} \otimes (\mathbf{J_C}\sigma_\alpha^2 + \mathbf{V}) + \sum_{i=1}^{r}\left((\mathbf{Z_i}\mathbf{Z_i}^T) \otimes \mathbf{J_C}\right)\sigma_i^2 + \mathbf{D}]\mathbf{M}||_F \\
=& argmin_{\boldsymbol{\theta}} ||(\mathbf{M}\mathbf{y}\mathbf{y}^T\mathbf{M} - \mathbf{MDM}) - \mathbf{M}[\mathbf{I_N} \otimes (\mathbf{J_C}\sigma_\alpha^2 + \mathbf{V}) + \sum_{i=1}^{r}\left((\mathbf{Z_i}\mathbf{Z_i}^T) \otimes \mathbf{J_C}\right)\sigma_i^2]\mathbf{M}||_F
\end{aligned}$$

To solve this, rewrite it as a linear function of $\boldsymbol{\theta}$. For convenience, we define $[i]$ as the row or column indexes in a matrix corresponding to individual i, that is from $(i-1) \times C + 1$ to $i \times C$; we define $\mathbf{L_{m,n}}$ as a $C \times C$ matrix of zeros, except for the entry of (m, n), which equals to one.

$$\begin{aligned}
&\text{vec}\left(\mathbf{M}[\mathbf{I_N} \otimes (\mathbf{J_C}\sigma_\alpha^2 + \mathbf{V}) + \sum_{i=1}^{r}\left((\mathbf{Z_i}\mathbf{Z_i}^T) \otimes \mathbf{J_C}\right)\sigma_i^2]\mathbf{M}\right) \\
&= \text{vec}\left(\mathbf{M}(\mathbf{I_N} \otimes \mathbf{J_C})\mathbf{M}\right)\sigma_\alpha^2 + \text{vec}\left(\mathbf{M}(\mathbf{I_N} \otimes \mathbf{V})\mathbf{M}\right) + \sum_{i=1}^{r}\text{vec}\left(\mathbf{M}\left((\mathbf{Z_i}\mathbf{Z_i}^T) \otimes \mathbf{J_C}\right)\mathbf{M}\right)\sigma_i^2 \\
&= \text{vec}\left(\mathbf{M}(\mathbf{I_N} \otimes \mathbf{J_C})\mathbf{M}\right)\sigma_\alpha^2 + \text{vec}\left(\mathbf{M}(\mathbf{I_N} \otimes \sum_{m,n}^{C}\mathbf{V}_{m,n}\mathbf{L}_{m,n})\mathbf{M}\right) + \sum_{i=1}^{r}\text{vec}\left(\mathbf{M}\left((\mathbf{Z_i}\mathbf{Z_i}^T) \otimes \mathbf{J_C}\right)\mathbf{M}\right)\sigma_i^2 \\
&= \text{vec}\left(\mathbf{M}(\mathbf{I_N} \otimes \mathbf{J_C})\mathbf{M}\right)\sigma_\alpha^2 + \sum_{m,n}^{C}\text{vec}\left(\mathbf{M}(\mathbf{I_N} \otimes \mathbf{L}_{m,n})\mathbf{M}\right)\mathbf{V}_{m,n} + \sum_{i=1}^{r}\text{vec}\left(\mathbf{M}\left((\mathbf{Z_i}\mathbf{Z_i}^T) \otimes \mathbf{J_C}\right)\mathbf{M}\right)\sigma_i^2 \\
&= \mathbf{Q}\boldsymbol{\theta}
\end{aligned}$$

Now we use the standard OLS projection of $\text{vec}\left(\mathbf{M}\mathbf{y}\mathbf{y}^T\mathbf{M} - \mathbf{MDM}\right)$ onto the span of $\mathbf{Q}$ to estimate $\boldsymbol{\theta}$, with $\widehat{\boldsymbol{\theta}} = (\mathbf{Q}^T\mathbf{Q})^{-1}\mathbf{Q}^T\text{vec}\left(\mathbf{M}\mathbf{y}\mathbf{y}^T\mathbf{M} - \mathbf{MDM}\right)$.

Here, $\mathbf{Q}^T := \begin{bmatrix} \text{vec}\left(\mathbf{M}(\mathbf{I_N} \otimes \mathbf{J_C})\mathbf{M}\right)^T \\ \text{vec}\left(\mathbf{M}\left(\mathbf{I_N} \otimes \mathbf{L_{1,1}}\right)\mathbf{M}\right)^T \\ ... \\ \text{vec}\left(\mathbf{M}\left(\mathbf{I_N} \otimes \mathbf{L_{C,C}}\right)\mathbf{M}\right)^T \\ \text{vec}\left(\mathbf{M}\left(\mathbf{I_N} \otimes (\mathbf{L_{1,2}} + \mathbf{L_{2,1}})\right)\mathbf{M}\right)^T \\ ... \\ \text{vec}\left(\mathbf{M}\left(\mathbf{I_N} \otimes (\mathbf{L_{C-1,C}} + \mathbf{L_{C,C-1}})\right)\mathbf{M}\right)^T \\ \text{vec}\left(\mathbf{M}\left((\mathbf{Z_1}\mathbf{Z_1}^T) \otimes \mathbf{J_C}\right)\mathbf{M}\right)^T \\ ... \\ \text{vec}\left(\mathbf{M}\left((\mathbf{Z_r}\mathbf{Z_r}^T) \otimes \mathbf{J_C}\right)\mathbf{M}\right)^T \end{bmatrix}$.

In special cases, $\mathbf{Q}$ can be simplified. In Hom model where $\mathbf{V} = 0$, we have $\boldsymbol{\theta} = (\sigma_\alpha^2, \sigma_1^2, ..., \sigma_r^2)$ and $\mathbf{Q}^T = \begin{bmatrix} \text{vec}\left(\mathbf{M}(\mathbf{I_N} \otimes \mathbf{J_C})\mathbf{M}\right)^T \\ \text{vec}\left(\mathbf{M}\left((\mathbf{Z_1}\mathbf{Z_1}^T) \otimes \mathbf{J_C}\right)\mathbf{M}\right)^T \\ ... \\ \text{vec}\left(\mathbf{M}\left((\mathbf{Z_r}\mathbf{Z_r}^T) \otimes \mathbf{J_C}\right)\mathbf{M}\right)^T \end{bmatrix}$; in Free model where $\mathbf{V_{m,n}} = 0$ when $m \neq n$, we have $\boldsymbol{\theta} = (\sigma_\alpha^2, \mathbf{V}_{11}, ..., \mathbf{V}_{CC}, \sigma_1^2, ..., \sigma_r^2)$ and $\mathbf{Q}^T = \begin{bmatrix} \text{vec}\left(\mathbf{M}(\mathbf{I_N} \otimes \mathbf{J_C})\mathbf{M}\right)^T \\ \text{vec}\left(\mathbf{M}\left(\mathbf{I_N} \otimes \mathbf{L_{1,1}}\right)\mathbf{M}\right)^T \\ ... \\ \text{vec}\left(\mathbf{M}\left(\mathbf{I_N} \otimes \mathbf{L_{C,C}}\right)\mathbf{M}\right)^T \\ \text{vec}\left(\mathbf{M}\left((\mathbf{Z_1}\mathbf{Z_1}^T) \otimes \mathbf{J_C}\right)\mathbf{M}\right)^T \\ ... \\ \text{vec}\left(\mathbf{M}\left((\mathbf{Z_r}\mathbf{Z_r}^T) \otimes \mathbf{J_C}\right)\mathbf{M}\right)^T \end{bmatrix}$.

2.2 Efficient computation: No additional random effects

In this section, through linear algebra manipulation, we reduce computation complexity for ML and REML under the special case where there are no random effects (other than $\boldsymbol{\alpha}$ and $\boldsymbol{\Gamma}$).

In ML, the rate limiting steps are the calculation of $\ln|\mathbb{V}(\mathbf{y})|$ and $(\mathbf{y} - \mathbf{Xb})^T \mathbb{V}(\mathbf{y})^{-1}(\mathbf{y} - \mathbf{Xb})$; in REML, those are the calculation of $\ln|\mathbb{V}(\mathbf{y})|$, $\mathbf{X}^T\mathbb{V}(\mathbf{y})^{-1}\mathbf{X}$, $\mathbf{y}^T\mathbb{V}(\mathbf{y})^{-1}\mathbf{y}$, and $\mathbf{X}^T\mathbb{V}(\mathbf{y})^{-1}\mathbf{y}$. Since the general form for $(\mathbf{y} - \mathbf{Xb})^T\mathbb{V}(\mathbf{y})^{-1}(\mathbf{y} - \mathbf{Xb})$, $\mathbf{X}^T\mathbb{V}(\mathbf{y})^{-1}\mathbf{X}$, $\mathbf{y}^T\mathbb{V}(\mathbf{y})^{-1}\mathbf{y}$, and $\mathbf{X}^T\mathbb{V}(\mathbf{y})^{-1}\mathbf{y}$ is $\mathbf{B}^T\mathbb{V}(\mathbf{y})^{-1}\mathbf{F}$, where $\mathbf{B}$ and $\mathbf{F}$ are tall matrices with only one or a few columns, we only need to reduce the computation complexity of $\ln|\mathbb{V}(\mathbf{y})|$ and $\mathbf{B}^T\mathbb{V}(\mathbf{y})^{-1}\mathbf{F}$.

When there is no extra random effect factor, i.e., $r = 0$. Define $[i]$ as indices corresponding to individual i, and $\mathbf{D}_{[i][i]} := \text{diag}(\boldsymbol{\nu}_{i,})$. The variance matrix of $\mathbf{y}$ and its inverse can be simplified

$$\begin{aligned} \mathbb{V}(\mathbf{y}) =& \mathbf{I_N} \otimes (\mathbf{J_C}\sigma_\alpha^2 + \mathbf{V}) + \mathbf{D} \\ =& \oplus_{i=1}^N (\mathbf{J_C}\sigma_\alpha^2 + \mathbf{V} + \mathbf{D}_{[i][i]}) \\ \mathbb{V}(\mathbf{y})^{-1} =& \oplus_{i=1}^N \left(\mathbf{J_C}\sigma_\alpha^2 + \mathbf{V} + \mathbf{D}_{[i][i]}\right)^{-1} \end{aligned}$$

Here, the $\oplus$ is the diagnal matrix of blocks, that is $\mathbf{A} \oplus \mathbf{B} := \begin{bmatrix} \mathbf{A} & 0 \\ 0 & \mathbf{B} \end{bmatrix}$ and $\oplus_{i=1}^{2} \mathbf{A} := \begin{bmatrix} \mathbf{A} & 0 \\ 0 & \mathbf{A} \end{bmatrix}$. Therefore, we can efficiently calculate the terms in the likelihoods of ML and REML:

$$\begin{aligned} \ln |\mathbb{V}(\mathbf{y})| &= \ln | \oplus_{i=1}^{N} \left(\mathbf{J_C} \sigma_\alpha^2 + \mathbf{V} + \mathbf{D}_{[i][i]} \right) | \\ &= \ln \prod_{i=1}^{N} |\mathbf{J_C} \sigma_\alpha^2 + \mathbf{V} + \mathbf{D}_{[i][i]}| \\ &= \sum_{i=1}^{N} \ln |\mathbf{J_C} \sigma_\alpha^2 + \mathbf{V} + \mathbf{D}_{[i][i]}| \end{aligned}$$

$$\begin{aligned} \mathbf{B}^T \mathbb{V}(\mathbf{y})^{-1} \mathbf{F} &= \mathbf{B}^T \left(\oplus_{i=1}^{N} \left(\mathbf{J_C} \sigma_\alpha^2 + \mathbf{V} + \mathbf{D}_{[i][i]} \right)^{-1} \right) \mathbf{F} \\ &= \sum_{i=1}^{N} \mathbf{B}_{[i]}^T \left(\mathbf{J_C} \sigma_\alpha^2 + \mathbf{V} + \mathbf{D}_{[i][i]} \right)^{-1} \mathbf{F}_{[i]} \end{aligned}$$

Therefore, the computation complexity reduces from $O(N^3C^3)$ to $O(NC^3)$ for ML and REML.

2.3 Efficient computation: One additional random effects

When there is only one extra random effect factor, i.e., $r = 1$, and each individual belongs to only one level of the factor, i.e. for each row of $\mathbf{Z_1}$, there is only one element of 1 and all others are 0. Assuming the matrix $\mathbf{Z_1}$ has K levels and is ordered by levels, and each level k has x_k individuals, so that

$$\mathbf{Z_1} = \oplus_{k=1}^{K} \mathbf{1_{x_k}} = \begin{bmatrix} \mathbf{1_{x_1}} & & \dots & \\ & \mathbf{1_{x_2}} & \dots & \\ \dots & \dots & \dots & \dots \\ & & \dots & \mathbf{1_{x_K}} \end{bmatrix}$$

Here, $\mathbf{1_{x_k}}$ is a vector of 1 of length x_k. Then, $\mathbf{Z_1}\mathbf{Z_1}^T = \oplus_{k=1}^{K} \mathbf{J_{x_k}}$, where $\mathbf{J_{x_k}}$ is a matrix of 1 of shape $x_k \times x_k$. Define $\{k\}$ as row or column indexes corresponding to individuals in level k. The variance of $\mathbf{y}$ and its inverse can be simplified

$$\begin{aligned} \mathbb{V}(\mathbf{y}) &= \mathbf{I_N} \otimes (\mathbf{J_C} \sigma_\alpha^2 + \mathbf{V}) + [(\mathbf{Z_1}\mathbf{Z_1}^T) \otimes \mathbf{J_C}] \sigma_1^2 + \mathbf{D} \\ &= \oplus_{k=1}^{K} \left(\mathbf{I_{x_k}} \otimes (\mathbf{J_C} \sigma_\alpha^2 + \mathbf{V}) \right) + [(\oplus_{k=1}^{K} \mathbf{J_{x_k}}) \otimes \mathbf{J_C}] \sigma_1^2 + \mathbf{D} \\ &= \oplus_{k=1}^{K} \left(\mathbf{I_{x_k}} \otimes (\mathbf{J_C} \sigma_\alpha^2 + \mathbf{V}) \right) + \oplus_{k=1}^{K} (\mathbf{J_{x_k}} \otimes \mathbf{J_C} \sigma_1^2) + \mathbf{D} \\ &= \oplus_{k=1}^{K} \left(\mathbf{I_{x_k}} \otimes (\mathbf{J_C} \sigma_\alpha^2 + \mathbf{V}) + \mathbf{J_{x_k \times C}} \sigma_1^2 + \mathbf{D}_{\{k\}\{k\}} \right) \\ \mathbb{V}(\mathbf{y})^{-1} &= \oplus_{k=1}^{K} \left(\mathbf{I_{x_k}} \otimes (\mathbf{J_C} \sigma_\alpha^2 + \mathbf{V}) + \mathbf{J_{x_k \times C}} \sigma_1^2 + \mathbf{D}_{\{k\}\{k\}} \right)^{-1} \end{aligned}$$

Therefore, we can efficiently calculate the terms in the likelihoods of ML and REML:

$$\begin{aligned}\ln|\mathbb{V}(\mathbf{y})| &= \ln|\oplus_{k=1}^{K}\left(\mathbf{I}_{\mathbf{x_k}}\otimes(\mathbf{J_C}\sigma_\alpha^2+\mathbf{V})+\mathbf{J}_{\mathbf{x_k}\times\mathbf{C}}\sigma_1^2+\mathbf{D}_{\{k\}\{k\}}\right)| \\ &= \ln\prod_{k=1}^{K}|\left(\mathbf{I}_{\mathbf{x_k}}\otimes(\mathbf{J_C}\sigma_\alpha^2+\mathbf{V})+\mathbf{J}_{\mathbf{x_k}\times\mathbf{C}}\sigma_1^2+\mathbf{D}_{\{k\}\{k\}}\right)| \\ &= \sum_{k=1}^{K}\ln|\left(\mathbf{I}_{\mathbf{x_k}}\otimes(\mathbf{J_C}\sigma_\alpha^2+\mathbf{V})+\mathbf{J}_{\mathbf{x_k}\times\mathbf{C}}\sigma_1^2+\mathbf{D}_{\{k\}\{k\}}\right)|\end{aligned}$$

$$\begin{aligned}\mathbf{B}^T\mathbb{V}(\mathbf{y})^{-1}\mathbf{F} &= \mathbf{B}^T\left(\oplus_{k=1}^{K}\left(\mathbf{I}_{\mathbf{x_k}}\otimes(\mathbf{J_C}\sigma_\alpha^2+\mathbf{V})+\mathbf{J}_{\mathbf{x_k}\times\mathbf{C}}\sigma_1^2+\mathbf{D}_{\{k\}\{k\}}\right)^{-1}\right)\mathbf{F} \\ &= \sum_{k=1}^{K}\mathbf{B}_{\{k\},}^T\left(\mathbf{I}_{\mathbf{x_k}}\otimes(\mathbf{J_C}\sigma_\alpha^2+\mathbf{V})+\mathbf{J}_{\mathbf{x_k}\times\mathbf{C}}\sigma_1^2+\mathbf{D}_{\{k\}\{k\}}\right)^{-1}\mathbf{F}_{\{k\},}\end{aligned}$$

These manipulations reduce the computational complexity from $O(N^3C^3)$ to $O(KN_b^3C^3)$ for ML and REML, where N_b is the number of individuals per level.

3 Simulation

3.1 Variance partition of overall pseudobulk

We parameterize our simulations in terms of interpretable variance components of the OP data, which we partition using Eq 5 in the main text by:

$$\begin{aligned}\mathbb{V}(y) &= \mathbb{V}(\mathbb{E}(y_i))+\mathbb{E}(\mathbb{V}(y_i)) \\ &= \mathbb{V}(\mathbf{P}_{i,}\boldsymbol{\beta})+\mathbb{E}\left(\sigma_\alpha^2+\mathbf{P}_{i,}\mathbf{V}\mathbf{P}_{i,}^T+\boldsymbol{\nu}_i\right) \\ &= \boldsymbol{\beta}^T\mathbf{S}\boldsymbol{\beta}+\sigma_\alpha^2+\mathbb{E}\left(\mathbf{P}_{i,}\mathbf{V}\mathbf{P}_{i,}^T\right)+\mathbb{E}(\boldsymbol{\nu}_i) \\ &= \boldsymbol{\beta}^T\mathbf{S}\boldsymbol{\beta}+\sigma_\alpha^2+\mathbb{E}\left(\operatorname{tr}\left(\mathbf{P}_{i,}\mathbf{V}\mathbf{P}_{i,}^T\right)\right)+\mathbb{E}(\boldsymbol{\nu}_i) \\ &= \boldsymbol{\beta}^T\mathbf{S}\boldsymbol{\beta}+\sigma_\alpha^2+\operatorname{tr}\left(\mathbf{V}\mathbb{E}\left(\mathbf{P}_{i,}^T\mathbf{P}_{i,}\right)\right)+\mathbb{E}(\boldsymbol{\nu}_i) \\ &= \boldsymbol{\beta}^T\mathbf{S}\boldsymbol{\beta}+\sigma_\alpha^2+\operatorname{tr}\left(\mathbf{V}\left(\mathbf{S}+\boldsymbol{\pi}\boldsymbol{\pi}^T\right)\right)+\mathbb{E}(\boldsymbol{\nu}_i) \\ &= \underbrace{\boldsymbol{\beta}^T\mathbf{S}\boldsymbol{\beta}}_{\text{cell type-specific mean}}+\underbrace{\sigma_\alpha^2}_{\text{cell type-shared variation}}+\underbrace{\operatorname{tr}(\mathbf{VS})+\boldsymbol{\pi}^T\mathbf{V}\boldsymbol{\pi}}_{\text{cell type-specific variation}}+\underbrace{\mathbb{E}(\boldsymbol{\nu}_i)}_{\text{measurement noise}}\end{aligned} \tag{3}$$

Here, $\mathbf{S} := \frac{1}{N}\mathbf{P_d}^T\mathbf{P_d}$ is the covariance matrix for cell type proportions; $\mathbf{P_d} := \mathbf{P}-\mathbf{1_N}\boldsymbol{\pi}^T$ is demeaned cell type proportion matrix; $\boldsymbol{\pi}$ is the vector of mean cell type proportions. We partition the variance of OP into four components: cell type fixed effect, homogeneous random effect, cell type-specific random effect, and noise.

3.2 Simulation of overall pseudobulk and cell type-specific pseudobulk

We set parameters for our simulation according to the four variance components of OP in (3). In the simulation of Free model, we assumed each of the four components explained 25% of

variance, i.e., $\boldsymbol{\beta}^T \mathbf{S} \boldsymbol{\beta} = \sigma_\alpha^2 = \operatorname{tr}(\mathbf{VS}) + \boldsymbol{\pi}^T \mathbf{V} \boldsymbol{\pi} = \mathbb{E}(\boldsymbol{\nu}_i) = 0.25$. We simulated 100 individuals and 4 cell types. Cell type proportions for each individual ($\mathbf{P}_{i,}$) were i.i.d. sampled from Dirichlet distribution $Dir(2,2,2,2)$, so each cell type has an expected proportion of 25%, i.e., $\boldsymbol{\pi}^T = \begin{bmatrix} 0.25 & 0.25 & 0.25 & 0.25 \end{bmatrix}$. From Dirichlet distribution, we also calculate the covariance matrix ($\mathbf{S}$) of cell type proportions. Assuming the ratio of fixed effects for the four cell types is $\boldsymbol{\beta}_1 : \boldsymbol{\beta}_2 : \boldsymbol{\beta}_3 : \boldsymbol{\beta}_4 = 8 : 4 : 2 : 1$, we calculated the fixed effects for each cell type $\boldsymbol{\beta}$, with the constraint of $\boldsymbol{\beta}^T \mathbf{S} \boldsymbol{\beta} = 0.25$. Assuming the ratio of cell type-specific variances is $\mathbf{V}_{11} : \mathbf{V}_{22} : \mathbf{V}_{33} : \mathbf{V}_{44} = 64 : 16 : 4 : 1$, we calculated the cell type-specific variance matrix $\mathbf{V}$ with the constraint of $\operatorname{tr}(\mathbf{VS}) + \boldsymbol{\pi}^T \mathbf{V} \boldsymbol{\pi} = 0.25$. To simulate measurement noise, we assume that the residual effect $\boldsymbol{\epsilon}_{ics}$ for gene expression in s-th cell from cell type c in individual i is i.i.d. across all cells for each individual with $\mathbb{V}(\boldsymbol{\epsilon}_{ics}) = \sigma_i^2$, such that $\boldsymbol{\nu}_i = \frac{\sigma_i^2}{n_i}$, $\boldsymbol{\nu}_{ic} = \frac{\sigma_i^2}{n_{ic}}$, and $\boldsymbol{\nu}_{ic} = \frac{\boldsymbol{\nu}_i}{\mathbf{P}_{ic}}$. We sampled the noise variance for each individual from a Gamma distribution $\boldsymbol{\nu}_i \overset{\text{ind}}{\sim} \Gamma(k = \frac{25}{8}, \theta = 0.08)$, which has $\mathbb{E}(\boldsymbol{\nu}_i) = k\theta = 0.25$ and $\mathbb{V}(\boldsymbol{\nu}_i) = k\theta^2 = 0.02$. Then, we calculated $\boldsymbol{\nu}_{ic}$ using $\boldsymbol{\nu}_{ic} = \frac{\boldsymbol{\nu}_i}{\mathbf{P}_{ic}}$. With these parameters, we generated OP and CTP using Eq 5 and Eq 6 in the main text.

The simulation process for the Hom and Full models is similar to the Free model, except for the variance decomposition of OP. In the Hom model, since there is no cell type-specific variation in the model, we assumed that homogeneously shared variation accounts for 50% of the variance of OP, i.e., $\sigma_\alpha^2 = 0.5$. In the Full model, since $\mathbf{V}$ and σ_α^2 are not jointly identified, we set $\sigma_\alpha^2 = 0$ and cell type-specific variation explained OP variance to 50%, i.e., $\operatorname{tr}(\mathbf{VS}) + \boldsymbol{\pi}^T \mathbf{V} \boldsymbol{\pi} = 0.5$. To account for the correlation of cell type-specific random effect between cell types, we not only set the ratio of cell type-specific variances of $\mathbf{V}_{11} : \mathbf{V}_{22} : \mathbf{V}_{33} : \mathbf{V}_{44} = 1 : 1 : 1 : 1$, but also set a correlation of 0.9 between nearby cell types and a correlation of 0.7 or 0.5 for others.

To test the performance of CTMM in various situations, we varied one parameter at a time, including sample size, cell type proportions, and cell type-specific variances. We also varied the number of cell types C with $C = 4, 8, 12$. To maintain a constant n_{ic}, we adjusted n_i accordingly as we increased C. Specifically, when $C = 8$ or 12, n_i is doubled or tripled compared to the case when $C = 4$. To capture this adjustment, we sampled the noise variance from a Gamma distribution with $\mathbb{E}(\boldsymbol{\nu}_i) = 0.25 \times \frac{4}{C}$. We attributed the residual variance because of reduced variance explained by measurement noise to cell type-specific mean. A list of tested parameters for each model is available in Supplementary Table S2. For the simulation of the Hom and Free models, we fit the simulated OP and CTP data into the Hom and Free models with maximum likelihood (ML), restricted maximum likelihood (REML), and Haseman-Elston regression (HE). We then tested for cell type-specific variance with applicable Wald tests and likelihood-ratio tests (LRT), as described in the main text. For the simulation of the Full model, we fit the simulated OP and CTP data into the Full model with ML, REML, and HE but did not perform hypothesis testing due to the statistical and computational complexity. We ran 1,000 replicates for each set of parameters.

3.3 Simulation with noisy $\boldsymbol{\nu}$

As $\boldsymbol{\nu}_{ic}$ is unknown and is estimated from cell-to-cell variation in practice, we also performed simulations to assess CTMM's sensitivity to estimation errors in $\boldsymbol{\nu}_{ic}$. We only examined CTP in this simulation, as it's far more powerful. As the simulation focuses on CTMM's utility in our real data analysis, we simulated Hom and Free models using parameters estimated in the iPSCs data. For each simulation setting, we ran 1,000 replicates where each uses parameters estimated from a distinct gene in a predefined set of 1,000 randomly chosen genes.

In the simulation of the Hom model, for each gene, we chose its parameters (σ_α^2 and $\boldsymbol{\beta}$) to match

the real CTMM estimates from the iPSCs data (estimated using REML under the Free model). If $\sigma_\alpha^2 < 0$, we set it to 0. Using cell type proportions $\mathbf{P}$ and measurement noise variance $\boldsymbol{\nu}_{ic}$ from the iPSCs data, we generated CTP using Eq 6 in the main text. To incorporate estimation error in $\boldsymbol{\nu}_{ic}$, for each $\boldsymbol{\nu}_{ic}$, we draw x_{ic} i.i.d. from a $Beta(2, b)$ distribution and then add $+x_{ic}\boldsymbol{\nu}_{ic}$ or $-x_{ic}\boldsymbol{\nu}_{ic}$ before inputting $\boldsymbol{\nu}_{ic}$ to CTMM. To cover the distribution of estimation error in iPSCs data, we simulated $b = 20, 10, 5, 3, 2$, to get corresponding coefficients of variation of 0.11, 0.20, 0.33, 0.45, and 0.55 for $\boldsymbol{\nu}_{ic}$.

We simulated under the Free model with varying cell type-specific variances. We obtained the parameters σ_α^2 and $\boldsymbol{\beta}$ in the same way as in the simulation of the Hom model. We varied the cell type-specific variance for cell type 1 ($\mathbf{V}_{11}$) from 0.05 to 0.5 and fixed other cell type-specific variances to 0.1. Those values were chosen based on the distribution of estimated cell type-specific variances in the iPSC data to make the simulation more realistic. For simplicity, the Free model simulations always use $b = 5$ (the most realistic value) to add estimation error into $\boldsymbol{\nu}_{ic}$.

3.4 Simulation of single-cell gene expression

To investigate the impact of cell number and read depth on CTMM, we simulated single-cell gene expression based on raw read counts from iPSCs[1]. For each set of simulation parameters, we simulated 1,000 replicates using the same set of 1,000 randomly selected genes.

To simulate the Hom model for each gene g, we subset the data to cells from the single cell type exhibiting the highest expression of gene g (across all cells from all individuals). We only kept individuals that had more than 10 cells and had at least one read of gene g. We simulated new data by drawing reads x_{gs} for gene g in cell s from a Binomial(R_s, p_{gs}) distribution, where R_s is the total number of reads in cell s and p_{gs} is the proportion of reads in cell s from gene g. Then, we simulated each individual by randomly drawing n_i cells with replacement from the individual's simulated cells. We repeated the process of cell sampling 4 times to simulate 4 cell types. To vary read depth and cell numbers, we simulated the total number of reads $R'_s = aR_s$ and sampled $n'_i = bn_i$ cells. We evaluated $a = 0.01$, 0.1, 1, and 2, and $b = 0.5$, 1, and 2 (Supplementary Figure 11).

We then reconstructed the input to CTMM from the sampled cells. We conducted log-transformation $\log_2 \frac{x_{gs} * 10^6}{R'_s}$, without the size factor normalization used in the original study[1]. The CTP was calculated as the mean of transformed counts across all cells per (individual, cell type) pair. Regarding measurement noise $\boldsymbol{\nu}_{ic}$ for individual i and cell type c, we first calculated cell-to-cell variance σ_i^2 as the variance of transformed counts across the n_i simulated cells and then calculated $\boldsymbol{\nu}_{ic} = \frac{\sigma_i^2}{n'_i}$. As all simulated cell types per individual were drawn from the same pool of simulated cells and had the same number of cells, they had the same value of $\boldsymbol{\nu}_{ic}$. To simulate the estimation error of $\boldsymbol{\nu}_{ic}$, we used the same procedure as described in the previous section of Simulation with noisy $\boldsymbol{\nu}$.

To extend this framework to simulate the Free model, we permuted CTP_{ic} and corresponding $\boldsymbol{\nu}_{ic}$ among individuals independently for each cell type. In this way, we broke down the shared variance between cell types while retaining the total variance for each cell type. The permuted data is a special case of the Free model where all cell types had equal cell type-specific variance. To simulate various degrees of cell type-specific variance, we randomly selected a proportion (p) of individuals and conducted a permutation on them. We evaluated $p = 0$, 0.2, 0.4, 0.6, 0.8, or 1, which created cell type-specific variance of 0, 0.2, 0.4, 0.6, 0.8, or 1 for all cell types, while holding the sum of shared variance and cell type-specific variance to 1.

4 PBMCs analysis

In order to validate CTMM's performance on a large, droplet-based sequencing dataset, we analyzed the peripheral blood mononuclear cells (PBMCs) from Yazar *et al.*[2]. The dataset has been through a thorough process of quality control and consists of 1.27 million PMBCs from 982 donors and classified into 14 cell types: CD4+ Naive and Central Memory T cell ($CD4_{NC}$), CD4+ Effector Memory and Central Memory T cell ($CD4_{ET}$), CD4+ SOX4 T cell ($CD4_{SOX4}$), CD8+ Naive and Central Memory T cell ($CD8_{NC}$), CD8+ Effector Memory T cell ($CD8_{ET}$), CD8+ S100B T cell ($CD8_{S100B}$), Natural Killer cell (NK), Natural Killer cell Recruiting (NK_R), Immature and Naive B cell (B_{IN}), Memory B cell (B_{Mem}), Plasma cell (*Plasma*), Classical Monocyte ($Mono_C$), Non-Classical Monocyte ($Mono_{NC}$), and Dendritic cell (DC). For the one donor with technical replicates, we only included the replicate with the largest number of cells. For individual-cell type pairs with 10 or fewer cells, $\mathbf{y}_{ic}$ and $\boldsymbol{\nu}_{ic}$ were set to missing; requiring more than 10 cells is our general guidance, which we also did in the Cuomo *et al.* data. We filtered cell types with more than 10% of missing data, leaving 7 cell types including $CD4_{NC}$, $CD4_{ET}$, $CD8_{ET}$, $CD8_{NC}$, NK, B_{IN}, and B_{Mem}. We then excluded donors with any missing data, leaving 928 donors. We filtered genes expressed in less than 10% of the cohort within any cell type, leaving 11,526 genes. We normalized UMI (Unique Molecular Identifier) counts by $\log_{10}(UMI\ counts\ per\ 10,000 + 1)$ and then generated CTP expression data (i.e., $\mathbf{y}_{ic}$ and $\boldsymbol{\nu}_{ic}$). For each gene, we fit CTP expression data into the Free model with HE after standardizing OP to mean 0 and variance 1. We adjusted for sex, age, and the first principal component of OP expression. We have binned the cohort into 5-year interval groups and distinct groups for individuals below 25 and those exceeding 90 years of age. We also modeled experimental batches as a random effect.

To test the robustness of CTMM on rare cell types, we performed a secondary analysis. We adopted the same procedure as in the primary analysis, except we filtered cell types with more than 50% of missing data to include two rarer cell types, i.e., $Mono_C$ and $CD8_{S100B}$. After exc luding donors with missing data, 597 donors remained.

Supplementary Table 1. Prior LMM for scRNA-seq data

Paper	Pseudobulk or cell level	Cross-cell type variance model	Variance component test	Inference method
Tung 2017[3]	Cell	Hom*	No	blmer[4]
Martinez-Jimenez 2017[5]	Cell	Pseudo-Free**	Yes***	BASiCS[6]
Crowell 2020[7]	Cell	Hom	No^	bglmer[4]/lmer[8]/variancePartition[9]
Cuomo 2020[10]	Cell	Hom	No	LIMIX[11]
Cuomo 2022[12]	Cell	Pseudo-Full**	No^^	CellRegMap[12]
CTMM	Pseudobulk	Free + Full	New jackknife test	New methods to fit REML and method-of-moments

*In the special case where cell types are experimental batches and each batch contains exactly one donor, the batch effect model is similar to the simplified Free model where all cell types have equal variance (Supplementary Figure 33). **We use "Pseudo" for models that cannot decompose interindividual variation: "Pseudo-Free" omits the Hom component because cell types are each studied separately; "Pseudo-Full" assumes a pre-defined covariance across cell types that is identical for all genes. ***Applies to N=1 individual at a time. ^The "differential state" test does not distinguish cell type-specific expression in mean vs variance (x- vs y-axes in Figure 4A). ^^CellRegMap is a method to estimate and test eQTLs and does not apply to the variance components in CTMM.

Supplementary Table 2. Parameters used in simulations of the Hom, Free, and Full models.

Model	Sample size	Dirichlet distribution of cell type proportions	Proportions of variances explained by the four components	Ratio of cell type-specific random effect variance	Ratio of cell type-specific mean	Correlation of cell type-specific random effect
Hom (baseline)	100	(2, 2, 2, 2)	(.25, .5, 0, .25)	NA	8:4:2:1	NA
Hom	20	-	-	-	-	-
Hom	50	-	-	-	-	-
Hom	300	-	-	-	-	-
Hom	500	-	-	-	-	-
Hom	1,000	-	-	-	-	-
Hom	-	(0.666, 2, 2, 2)	-	-	-	-
Hom	-	(1.05, 2, 2, 2)	-	-	-	-
Hom	-	(4, 2, 2, 2)	-	-	-	-
Hom	-	(2,2,2,2,2,2,2,2)	(.375, .5, 0, .125)	-	8:8:4:4:2:2:1:1	-
Hom	-	(2,2,2,2,2,2, 2,2,2,2,2,2)	(.42, .5, 0, .08)	-	8:8:8:4:4:4:2:2:2:1:1:1	-
Free (baseline)	100	(2, 2, 2, 2)	(.25, .25, .25, .25)	8:4:2:1	8:4:2:1	NA
Free	20	-	-	-	-	-
Free	50	-	-	-	-	-
Free	300	-	-	-	-	-
Free	500	-	-	-	-	-
Free	1,000	-	-	-	-	-
Free	-	(0.666, 2, 2, 2)	-	-	-	-
Free	-	(1.05, 2, 2, 2)	-	-	-	-
Free	-	(4, 2, 2, 2)	-	-	-	-
Free	-	-	(.25, .10, .40, .25)	-	-	-
Free	-	-	(.25, .20, .30, .25)	-	-	-
Free	-	-	(.25, .30, .20, .25)	-	-	-
Free	-	-	(.25, .40, .10, .25)	-	-	-
Free	-	-	-	27:9:3:1	-	-
Free	-	-	-	2:1:1:1	-	-
Free	-	-	-	1:1:1:1	-	-
Free	-	(2,2,2,2,2,2,2,2)	(.375, .25, .25, .125)	8:8:4:4:2:2:1:1	8:8:4:4:2:2:1:1	-
Free	-	(2,2,2,2,2,2, 2,2,2,2,2,2)	(.42, .25, .25, .08)	8:8:8:4:4:4:2:2:2:1:1:1	8:8:8:4:4:4:2:2:2:1:1:1	-
Full (baseline)	100	(2, 2, 2, 2)	(.25, .25, .25, .25)	1:1:1:1	8:4:2:1	CT1-CT2, CT2-CT3, CT3-CT4: 0.9 CT1-CT3, CT2-CT4: 0.7 CT1-CT4: 0.5
Full	20	-	-	-	-	-
Full	50	-	-	-	-	-
Full	300	-	-	-	-	-
Full	500	-	-	-	-	-
Full	1,000	-	-	-	-	-

'-': indicates the same parameter as in the baseline model

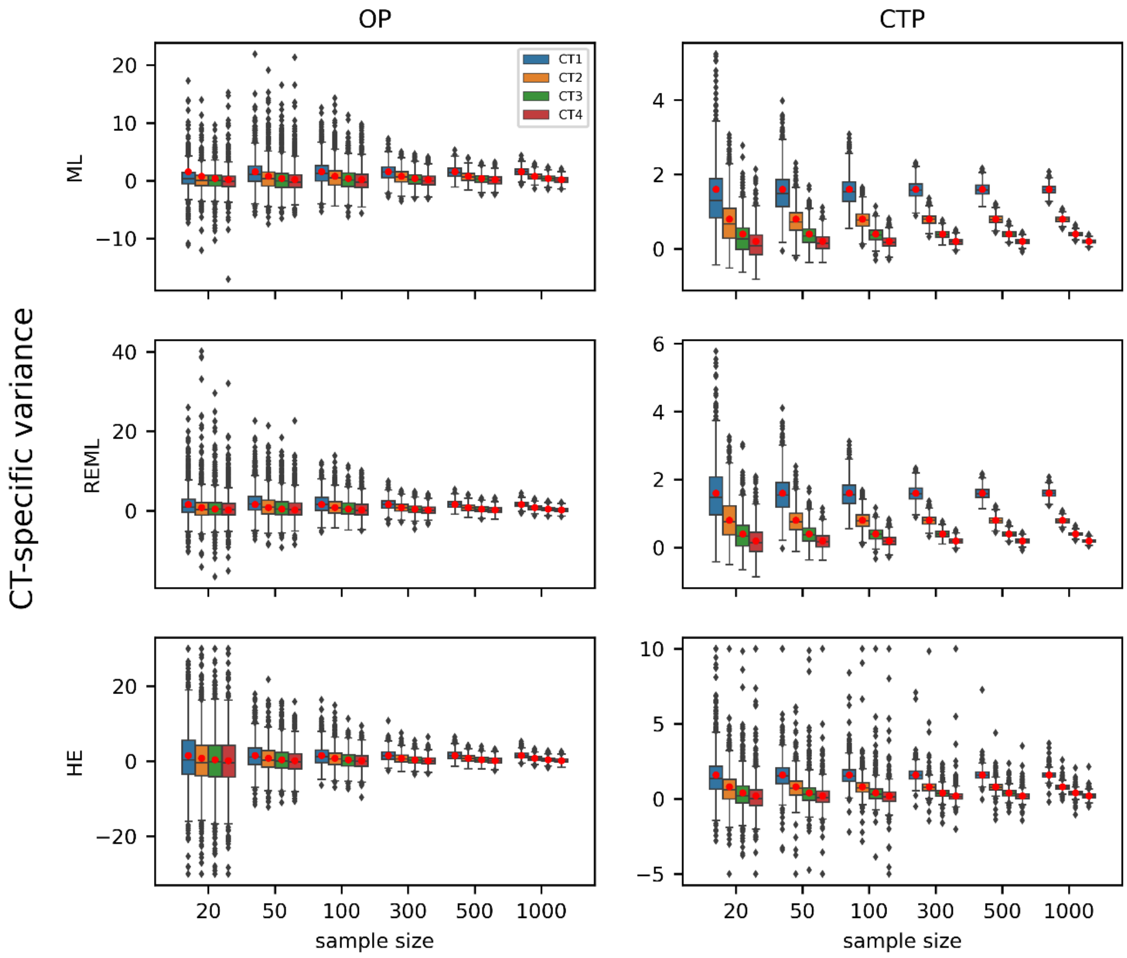

Supplementary Figure 1. CTMM estimates of cell type-specific variance with varying sample sizes. Estimates are fit under the Free model, and red dots indicate the true cell type-specific variances. Rows show different estimation methods. Columns show different inputs to CTMM, either overall pseudobulk (OP) or cell type-specific pseudobulk (CTP). Box plots indicate the distribution of estimated cell type-specific variances across 1,000 replicate simulations. Values were truncated to (-30, 30) in HE with OP and to (-5, 10) in HE with CTP. Box plots show the median and the first and third quartiles. The whiskers extend to values within 1.5 times the interquartile range from the first and third quartiles.

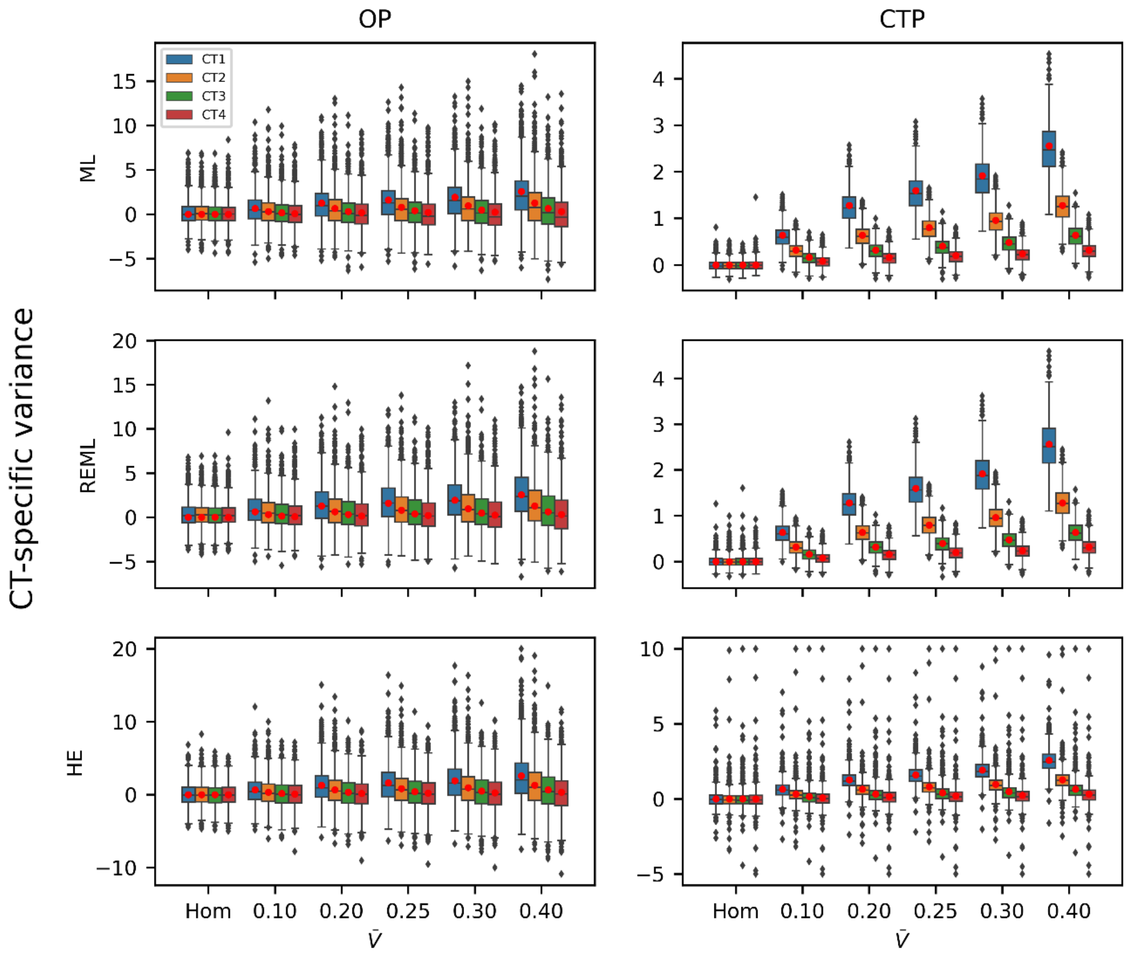

Supplementary Figure 2. CTMM estimates of cell type-specific variance with varying levels of true cell type-specific variance. $\bar{V}$ can be interpreted as the proportion of variance in the overall pseudobulk due to cell type-specific interindividual variation; the Hom model of no cell type-specificity is obtained when $\bar{V}$=0. Estimates are fit under the Free model, and red dots indicate the true cell type-specific variances. Rows show different estimation methods. Columns show different inputs to CTMM, either overall pseudobulk (OP) or cell type-specific pseudobulk (CTP). Box plots indicate the distribution of estimated cell type-specific variances across 1,000 replicate simulations. In HE with OP, values above 20 were truncated; in HE with CTP, values were truncated to (-5, 10). Box plots show the median and the first and third quartiles. The whiskers extend to values within 1.5 times the interquartile range from the first and third quartiles.

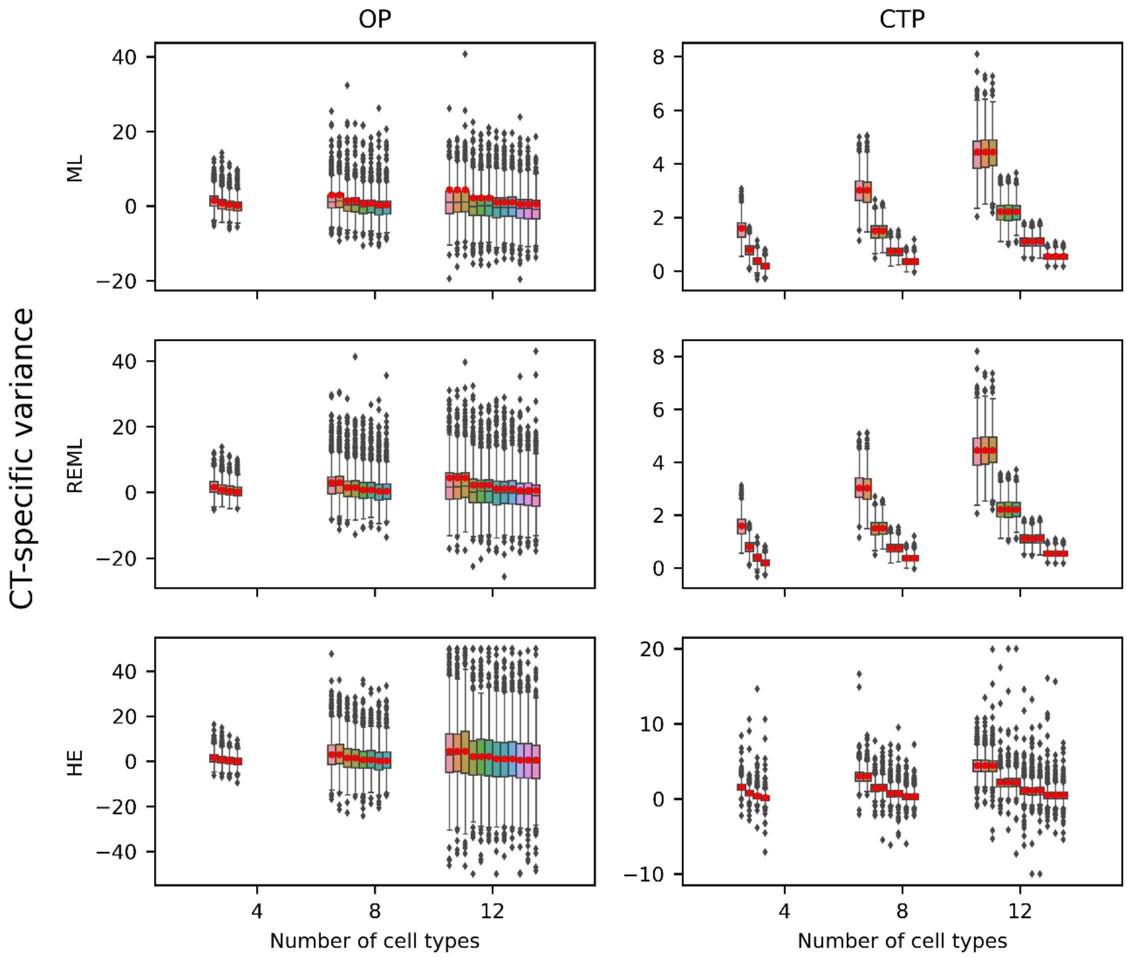

Supplementary Figure 3. CTMM estimates of cell type-specific variance with varying numbers of cell types. Estimates are fit under the Free model, and red dots indicate the true cell type-specific variances. Rows show different estimation methods. Columns show different inputs to CTMM, either overall pseudobulk (OP) or cell type-specific pseudobulk (CTP). Box plots indicate the distribution of estimated cell type-specific variances across 1,000 replicate simulations. Values were truncated to (-50, 50) in HE with OP and to (-10, 20) in HE with CTP. Box plots show the median and the first and third quartiles. The whiskers extend to values within 1.5 times the interquartile range from the first and third quartiles.

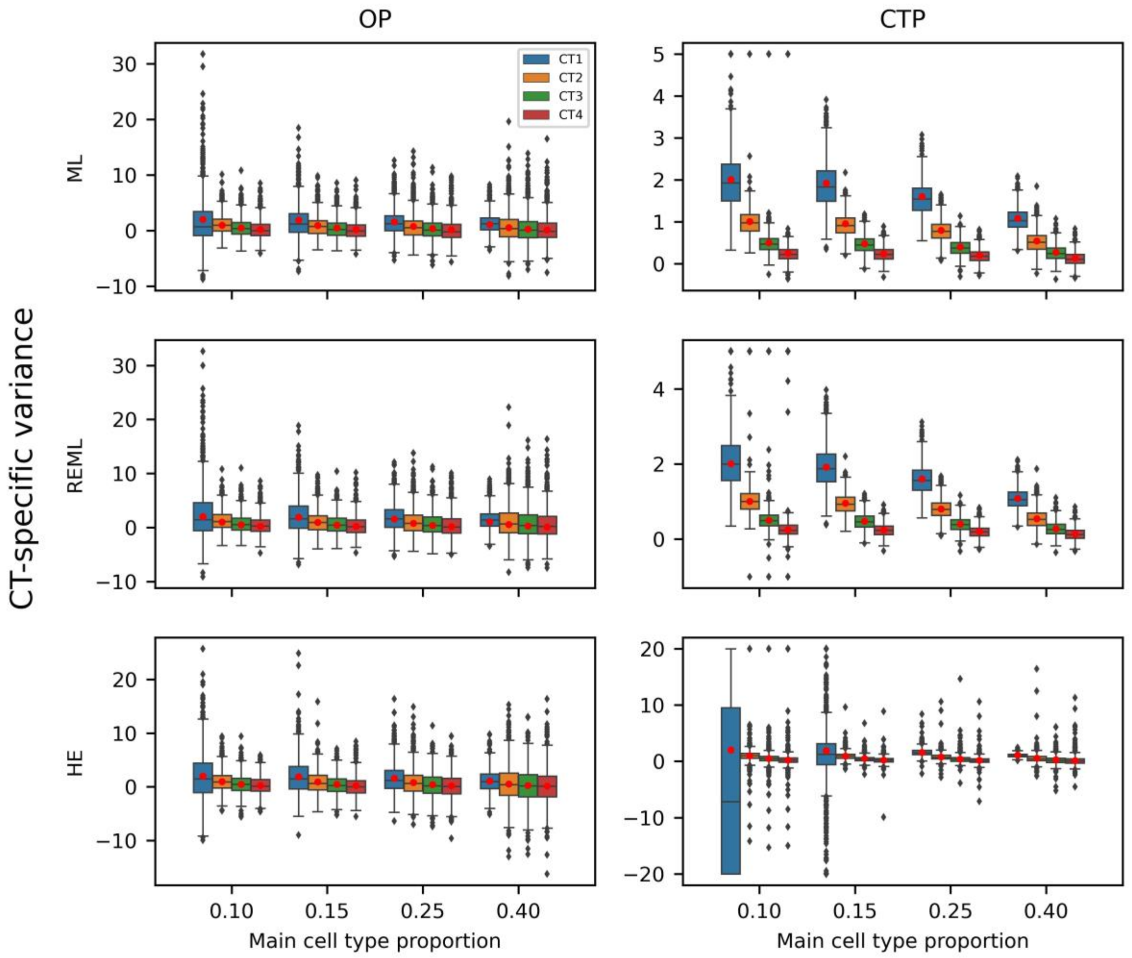

Supplementary Figure 4. CTMM estimates of cell type-specific variance with varying cell type proportions. The proportion of the "main" cell type, which has the largest cell type-specific variance, is varied, with the proportions of other cell types scaled so the total proportions sum to 1. Estimates are fit under the Free model, and red dots indicate the true cell type-specific variances. Rows show different estimation methods. Columns show different inputs to CTMM, either overall pseudobulk (OP) or cell type-specific pseudobulk (CTP). Box plots indicate the distribution of estimated cell type-specific variances across 1,000 replicate simulations. In ML with CTP, values above 5 were truncated; in REML with CTP, values were truncated to (-1, 5); in HE with CTP, values were truncated to (-20, 20). Box plots show the median and the first and third quartiles. The whiskers extend to values within 1.5 times the interquartile range from the first and third quartiles.

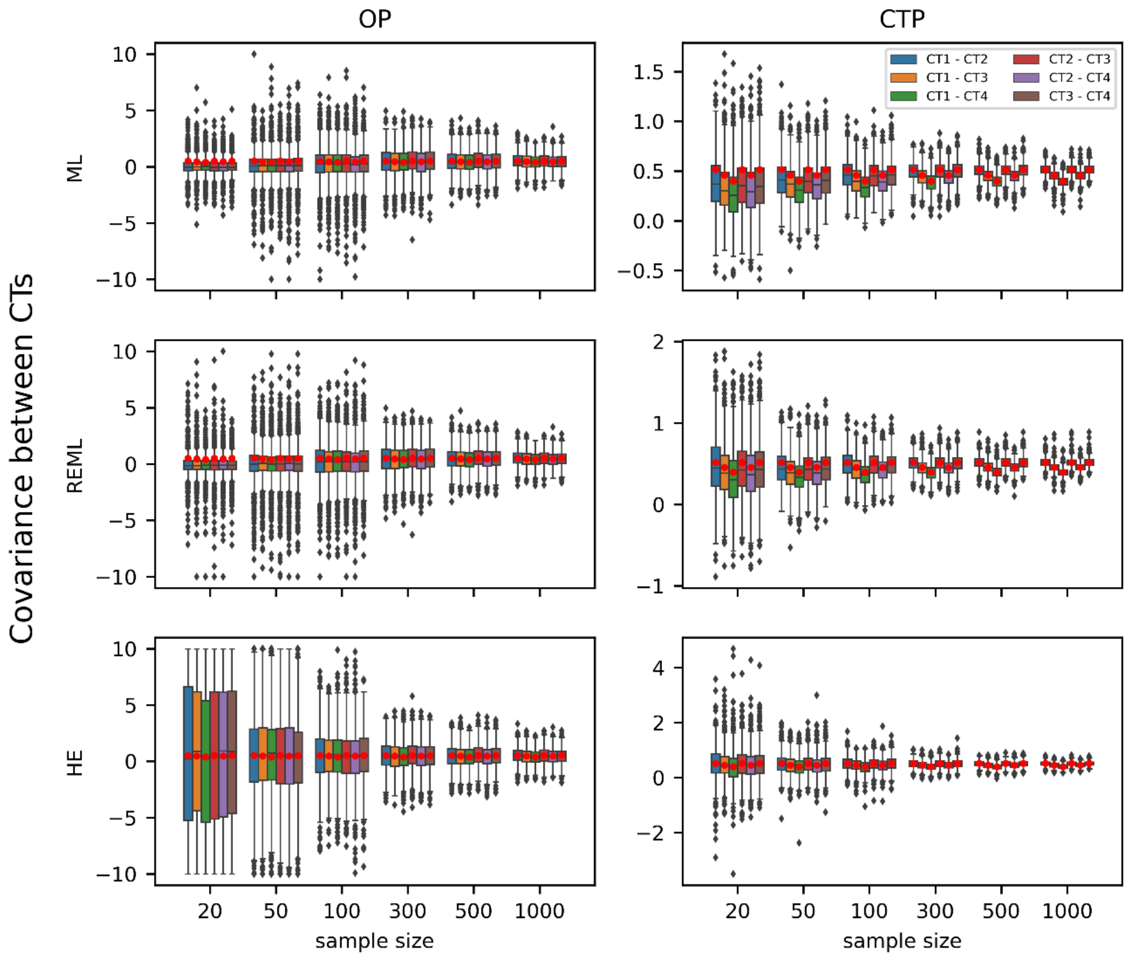

Supplementary Figure 5. CTMM estimates of cell type-specific covariance with varying sample sizes. Estimates are fit under the Full model, and red dots indicate the true cell type-specific covariances. Rows show different estimation methods. Columns show different inputs to CTMM, either overall pseudobulk (OP) or cell type-specific pseudobulk (CTP). Box plots indicate the distribution of estimated cell type-specific variances across 1,000 replicate simulations. In CTMM with OP, values were truncated to (-10, 10). Box plots show the median and the first and third quartiles. The whiskers extend to values within 1.5 times the interquartile range from the first and third quartiles.

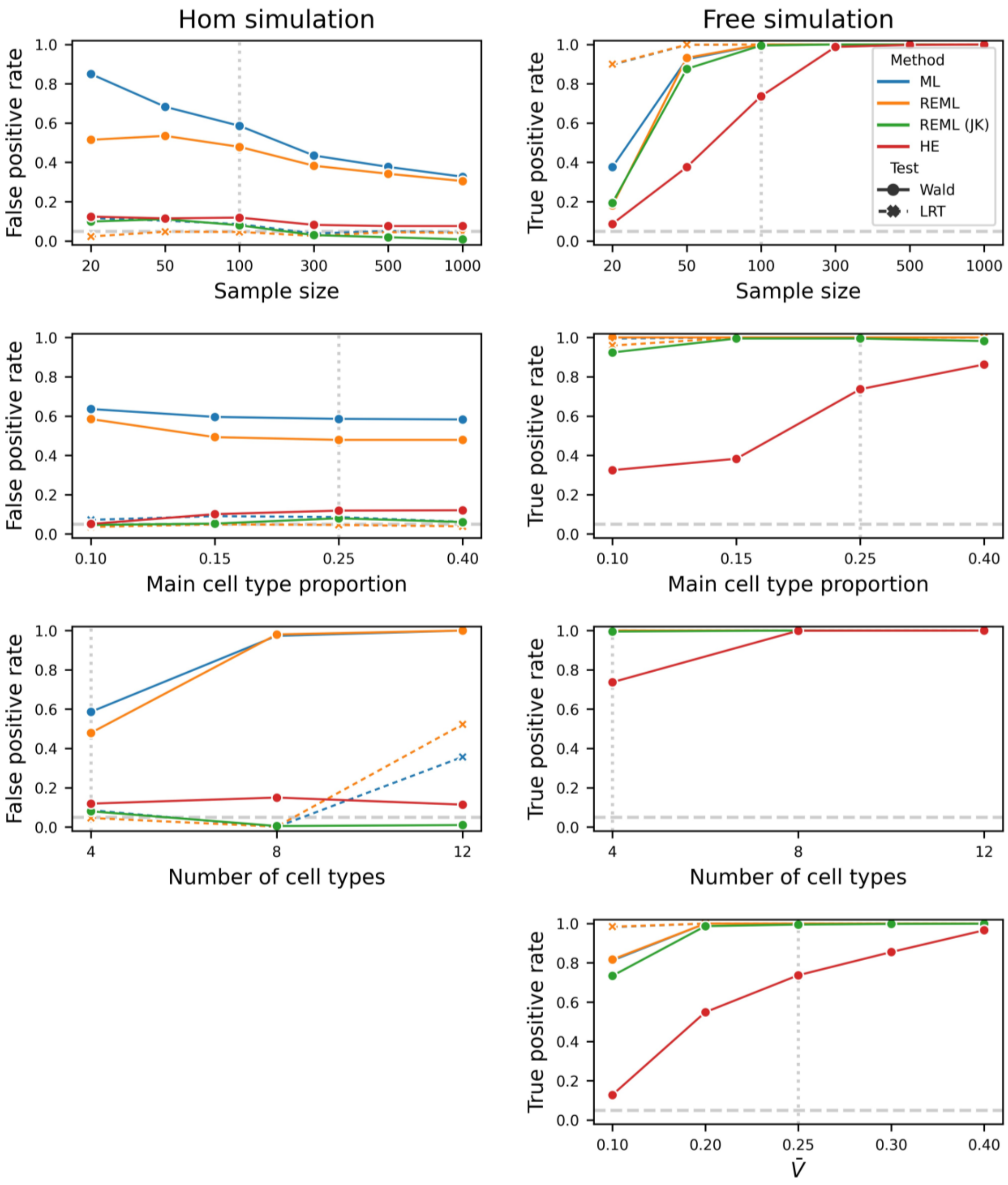

Supplementary Figure 6. Power of CTMM's test of cell type-specific variance with CTP data. The left column shows simulations under the null Hom model, where there is no cell type-specific variance. The right column shows simulations under the alternative Free model, where each cell type has its own cell type-specific variance. Vertical dashed lines indicate parameters used in the baseline models, as listed in Supplementary Table 2. Each row in order varies sample size (as in Supplementary Figure 2), cell type proportion (as in Supplementary Figure 5), number of cell types (as in Supplementary Figure 4), and $\bar{V}$, the proportion of overall variance explained by cell type-specific interindividual variation (as in Supplementary Figure 3).

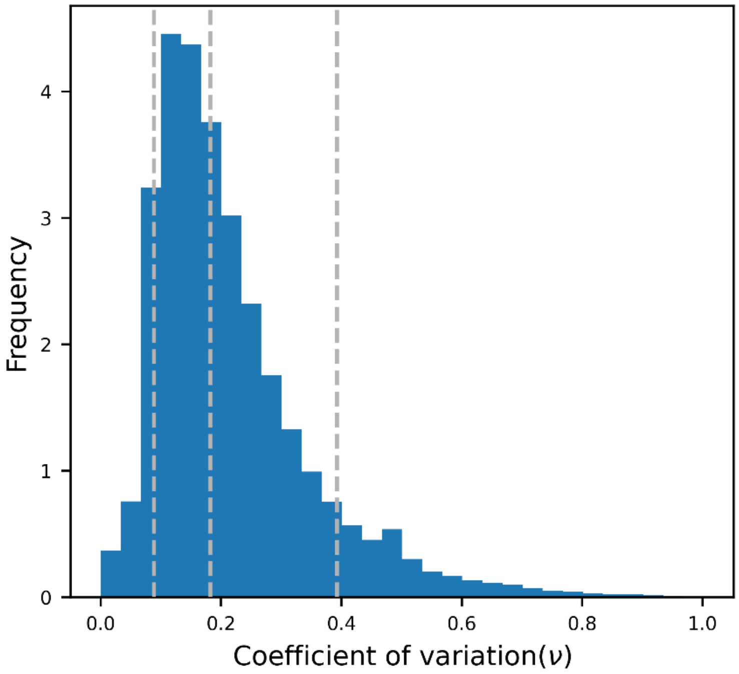

Supplementary Figure 7. Distribution of coefficient of variation for ν_{ic} across all combinations of individuals, cell types, and genes. Dashed lines indicate the 10%, 50%, and 90% percentiles.

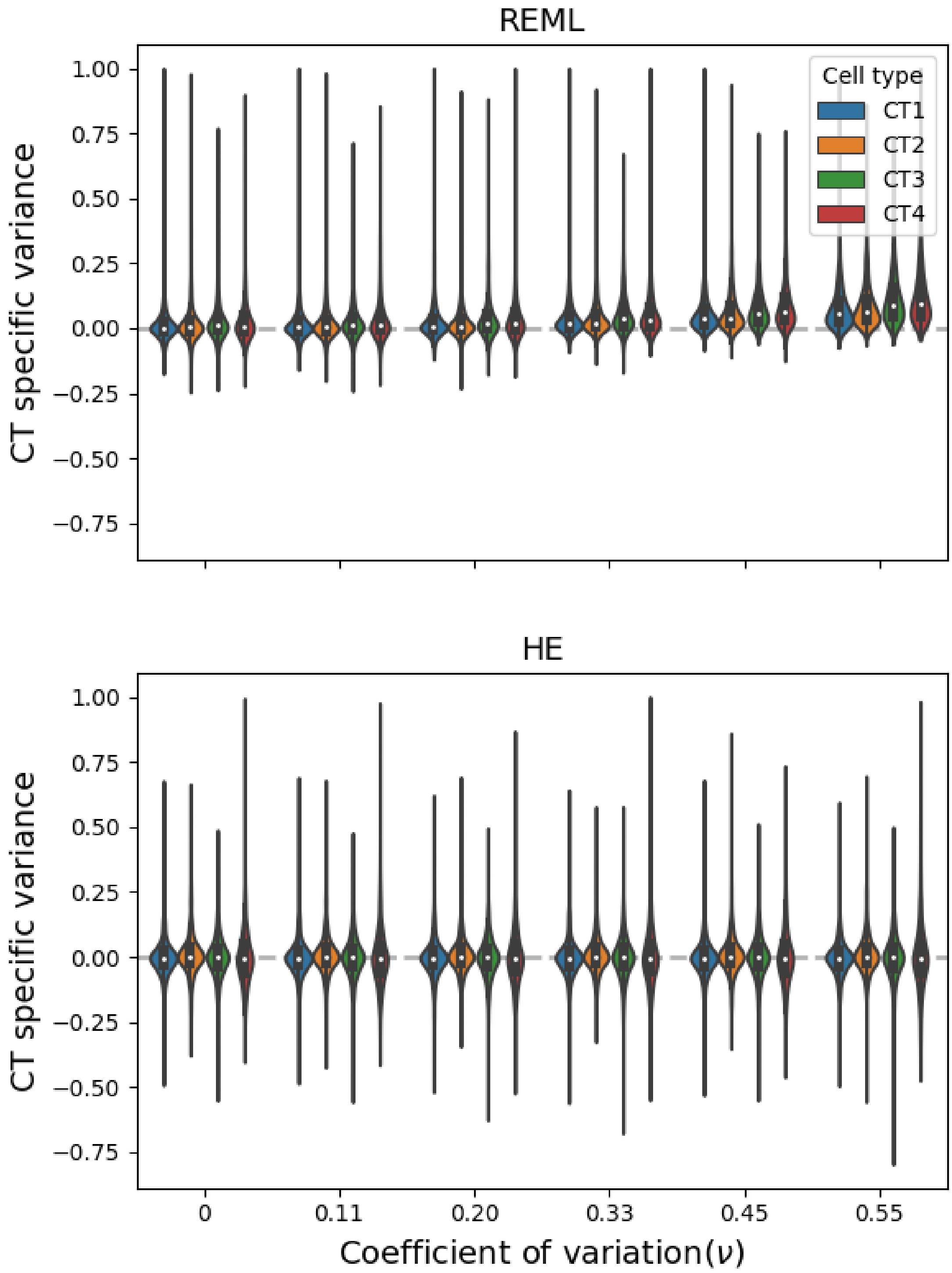

Supplementary Figure 8. CTMM estimates of cell type-specific variance in simulations of Hom model with noisy estimates of measurement error variance (ν_{ic}). The violin plots indicate the distribution of estimated cell type-specific variances across 1,000 replicate simulations. Values above 1 were truncated. Interior box plots show the median and the first and third quartiles. The whiskers extend to values within 1.5 times the interquartile range from the first and third quartiles.

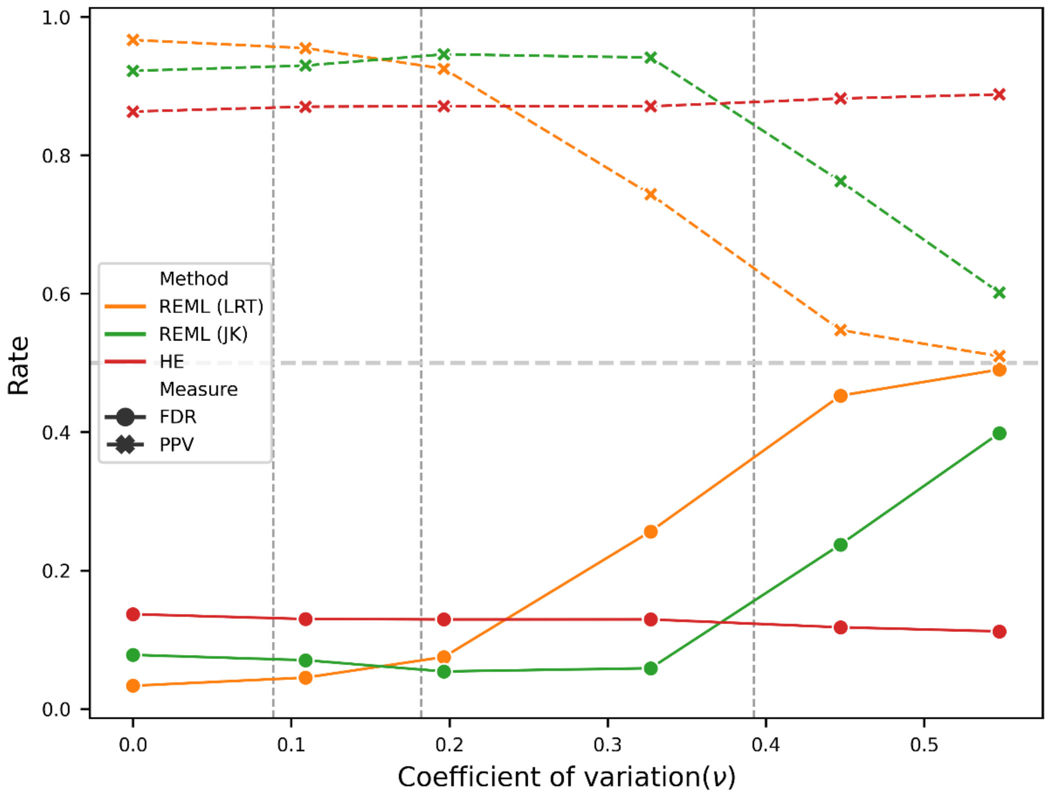

Supplementary Figure 9. Power of CTMM in simulations across varying levels of uncertainty in estimated noise variance (ν_{ic}). Vertical dashed lines indicate the 10%, 50%, and 90% percentiles of the transcriptome-wide distribution of coefficient of variation for ν_{ic} in the real iPSCs data. False discovery rate (FDR) and positive predictive value (PPV) were calculated with a 50:50 mixture of genes simulated from the Hom and Free models. In the simulation of the Free model, cell type-specific variance was equal to 0.1 for all cell types.

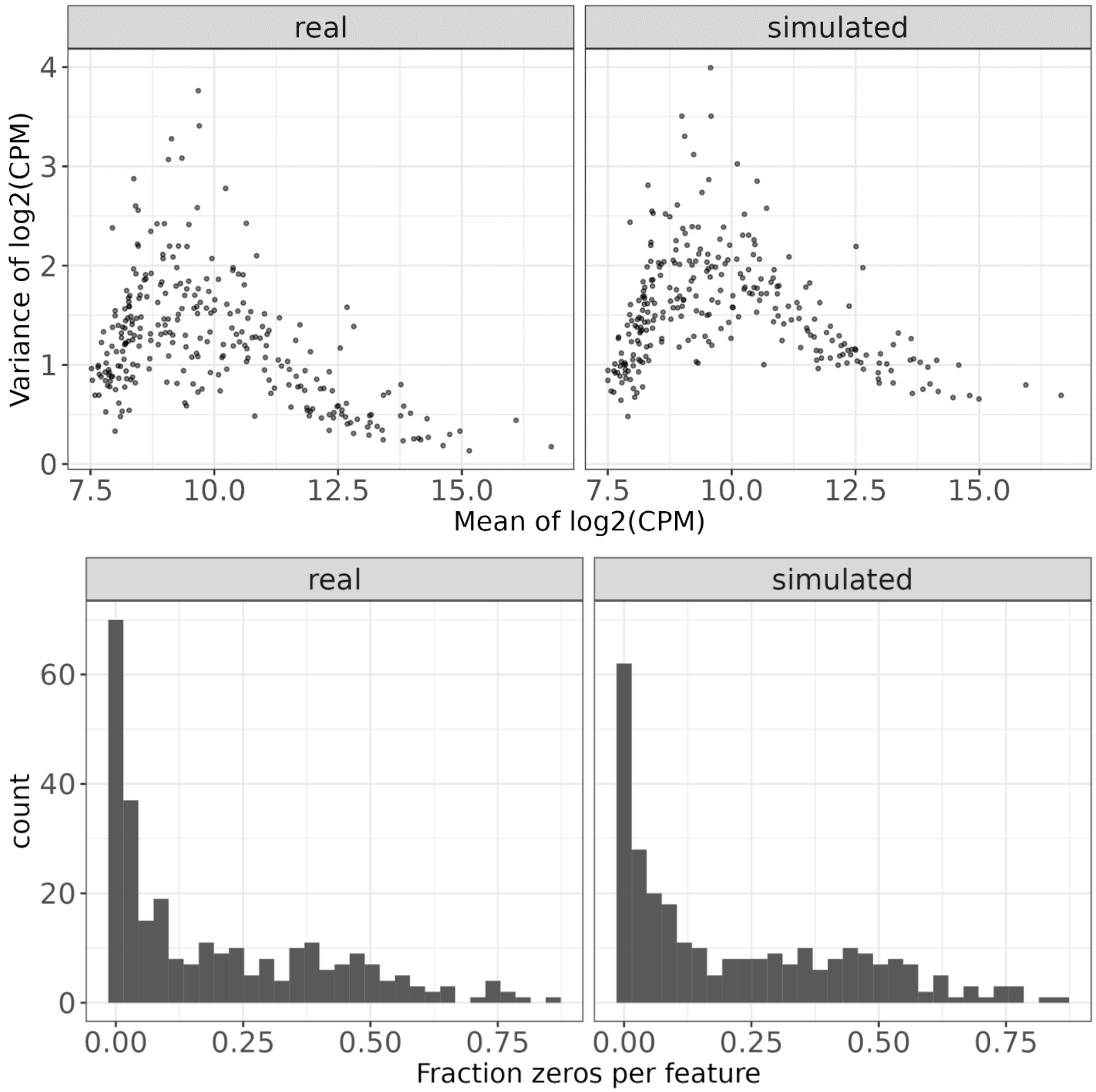

Supplementary Figure 10. Comparison of count distribution between real and simulated scRNA-seq data. The real data are genes from the iPSC data, and each simulated gene is based on parameters from one of these real genes (Supplementary Note 1 Section 3.4). We simulated 7202 cells, each corresponding to a real cell from the cell type “day0”. Each dot in the top panels shows a gene’s expression mean and variance across cells. The bottom panels show the distribution of the fraction of zeros per gene across the simulated genes.

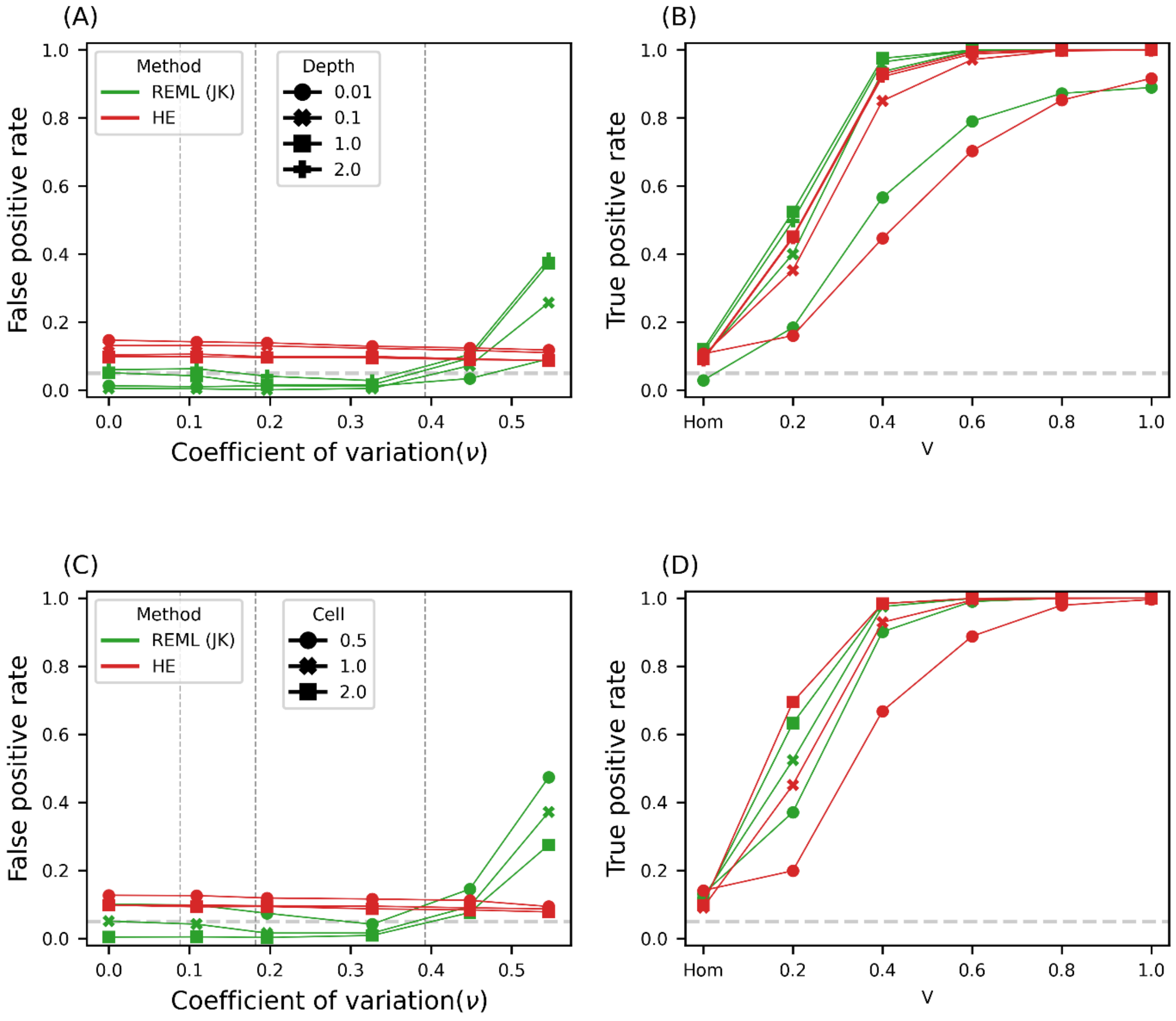

Supplementary Figure 11. Power of CTMM in single-cell gene expression simulations with various sequencing depths and numbers of cells. False positive rates under different levels of noise of $\boldsymbol{\nu}_{ic}$ when varying sequencing depth (A) and cell number (C). Dashed lines indicate the 10%, 50%, and 90% percentiles of the transcriptome-wide distribution of coefficient of variation for $\boldsymbol{\nu}_{ic}$ in the real iPSCs data. Analogous true positive rates using $Coefficient\ of\ variation(\boldsymbol{\nu}_{ic}) = 0.33$ as the cell type-specific variance (an equal number for all cell types) grows from 0 (Hom) to 1. Line types vary the sequencing depth (B) or cell number (D).

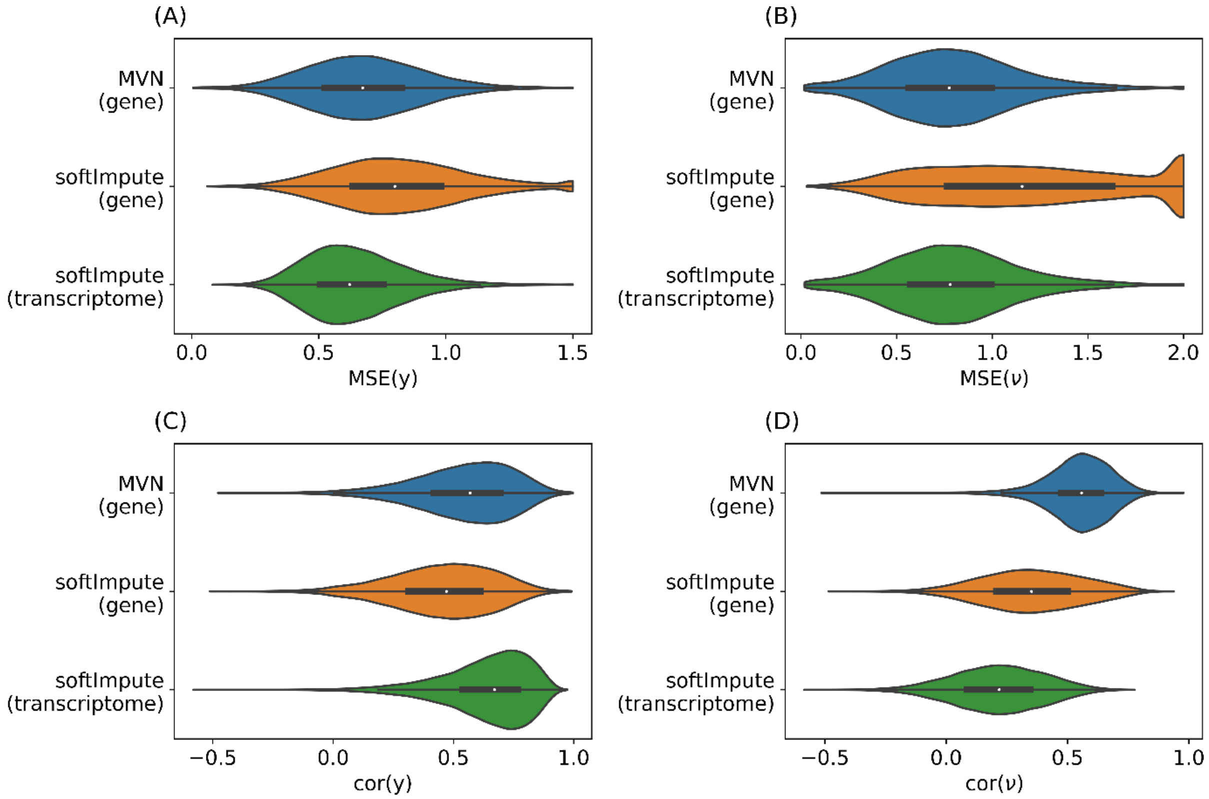

Supplementary Figure 12. Estimated imputation accuracies for cell type-specific pseudo-bulk and noise variance. Mean squared error (MSE) (A, B) and correlation (C, D) between raw and imputed cell type-specific pseudo-bulk (A, C) and noise variance (B, D). Each violin shows the distribution across the transcriptome of 11,231 genes. SoftImpute and MVN are two matrix imputation methods. “Gene” refers to imputing each gene separately; “transcriptome” refers to jointly imputing all genes. $MSE(y)$ was truncated at 1.5; $MSE(\nu)$ was truncated at 2. Interior box plots show the median and the first and third quartiles. The whiskers extend to values within 1.5 times the interquartile range from the first and third quartiles.

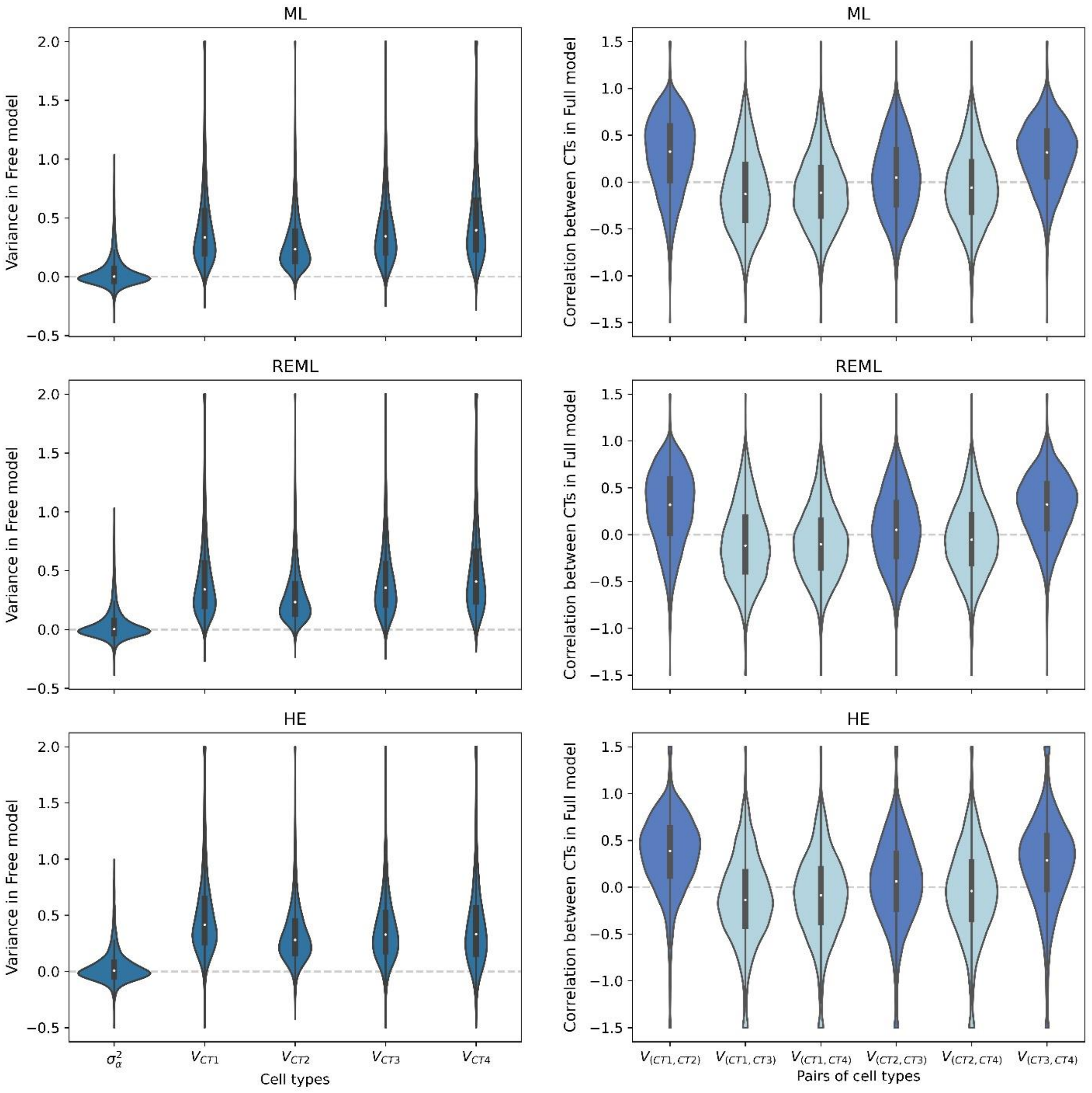

Supplementary Figure 13. Transcriptome-wide distribution of CTMM results with three estimation methods and CTP data. Left column shows results across 11,231 genes from the Free model: homogeneous variance (σ_α^2) and cell type-specific variances. Right column shows results from the Full model: correlation between each pair of cell types, with dark blue indicating adjacent cell type pairs and light blue indicating others. After removing genes with negative variances, ML, REML, and HE have 11,104, 11,122, and 10,032 genes left for correlation calculation. Homogeneous variance and cell type-specific variances were truncated to (-0.5,2); correlations between cell types were truncated to (-1.5,1.5). Interior box plots show the median and the first and third quartiles. The whiskers extend to values within 1.5 times the interquartile range from the first and third quartiles.

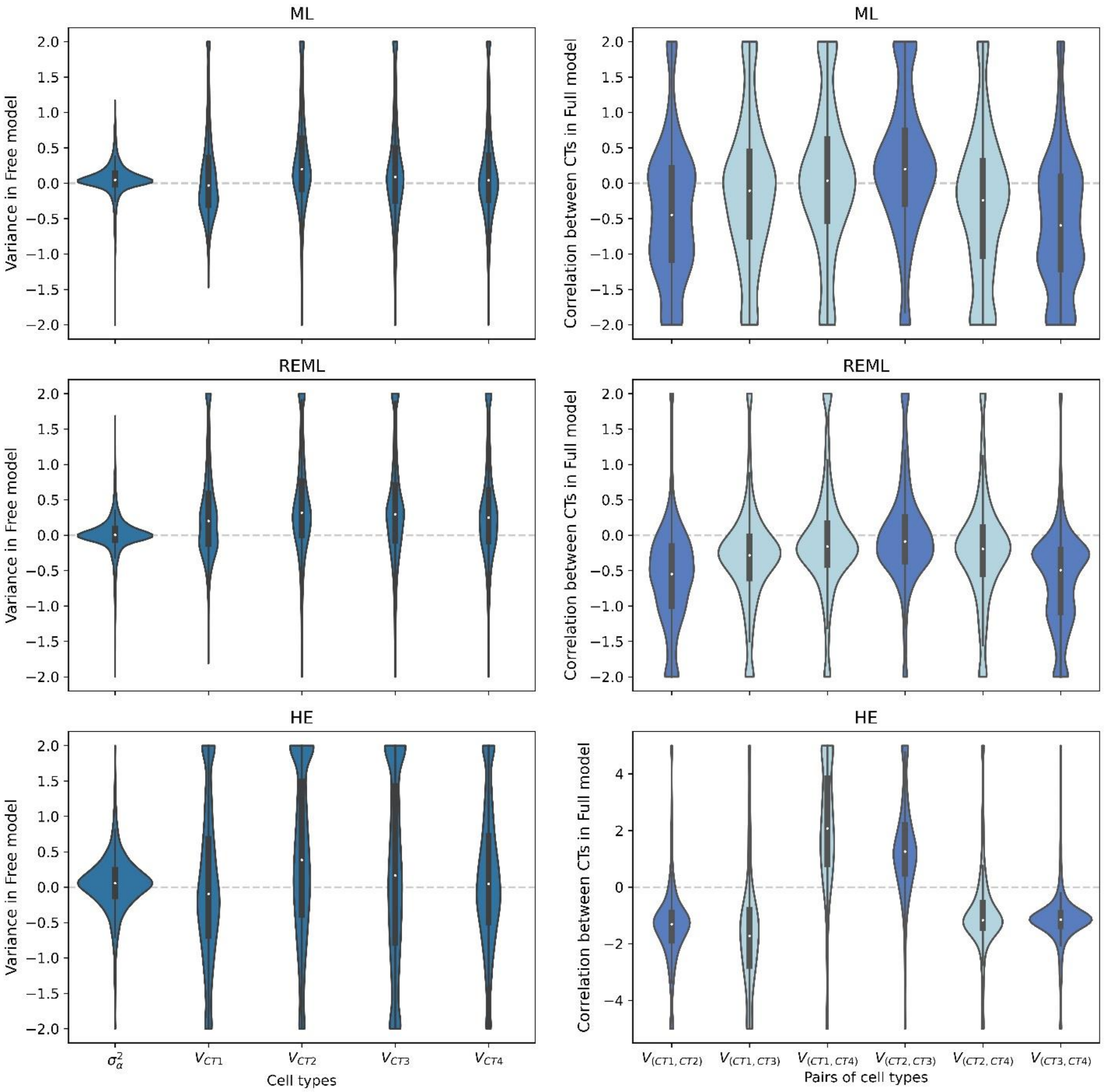

Supplementary Figure 14. Transcriptome-wide distribution of CTMM results with three estimation methods and OP data. Left column shows results across 11,231 genes from the Free model: homogeneous variance (σ_α^2) and cell type-specific variances. Right column shows results from the Full model: correlation between each pair of cell types, with dark blue indicating adjacent cell type pairs and light blue indicating others. After removing genes with negative variances, ML, REML, and HE have 1,457, 4,562, and 1,201 genes left for correlation calculation. Values were truncated to (-2, 2) except for the very noisy bottom-right panel which was truncated to (-5, 5). Interior box plots show the median and the first and third quartiles. The whiskers extend to values within 1.5 times the interquartile range from the first and third quartiles.

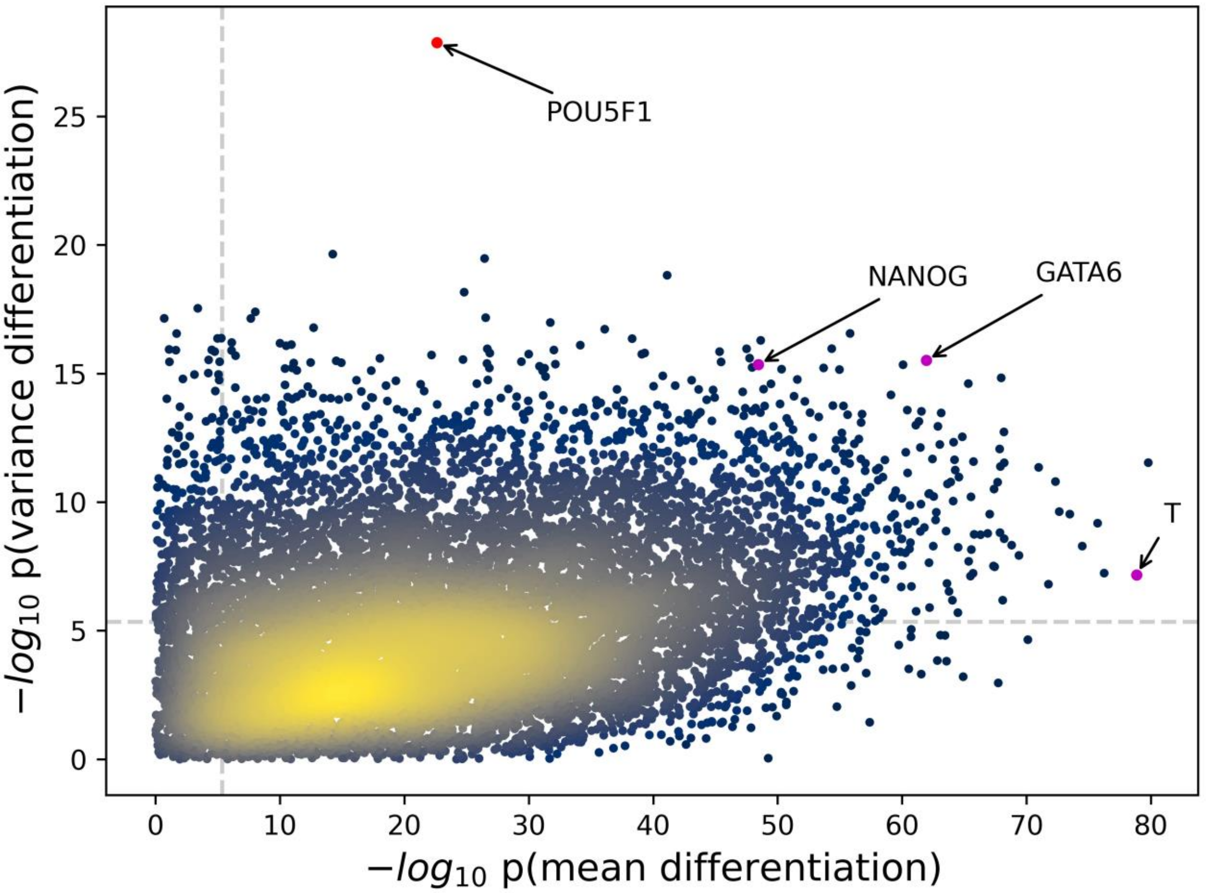

Supplementary Figure 15. Transcriptome-wide distribution of *p* values for differentiation in expression mean and variance using HE and CTP data. Each dot represents a gene. Dots are colored by the density of genes in the area, with yellow indicating denser distribution. Dashed lines indicate the significance threshold after Bonferroni correction. The three purple dots indicate the three maker genes used in *Cuomo et al*. to indicate each stem cell differentiation stage, spanning iPSC (*NANOG*), mesendoderm (*T*), and definitive endoderm (*GATA6*). The red dot indicates the top signal *POU5F1* found here and in REML with jackknife, which is one of the three core regulators in cell pluripotency.

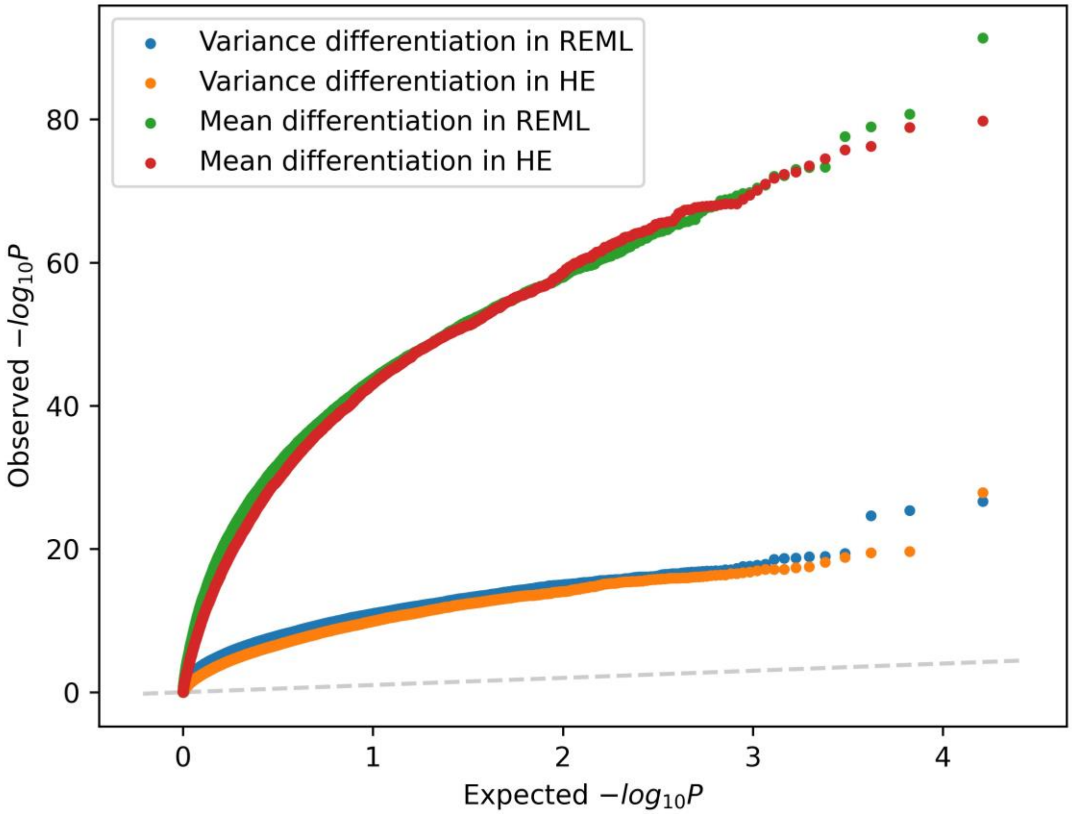

Supplementary Figure 16. Quantile-quantile plot of p-value distributions of variance differentiation and mean differentiation in REML and HE.

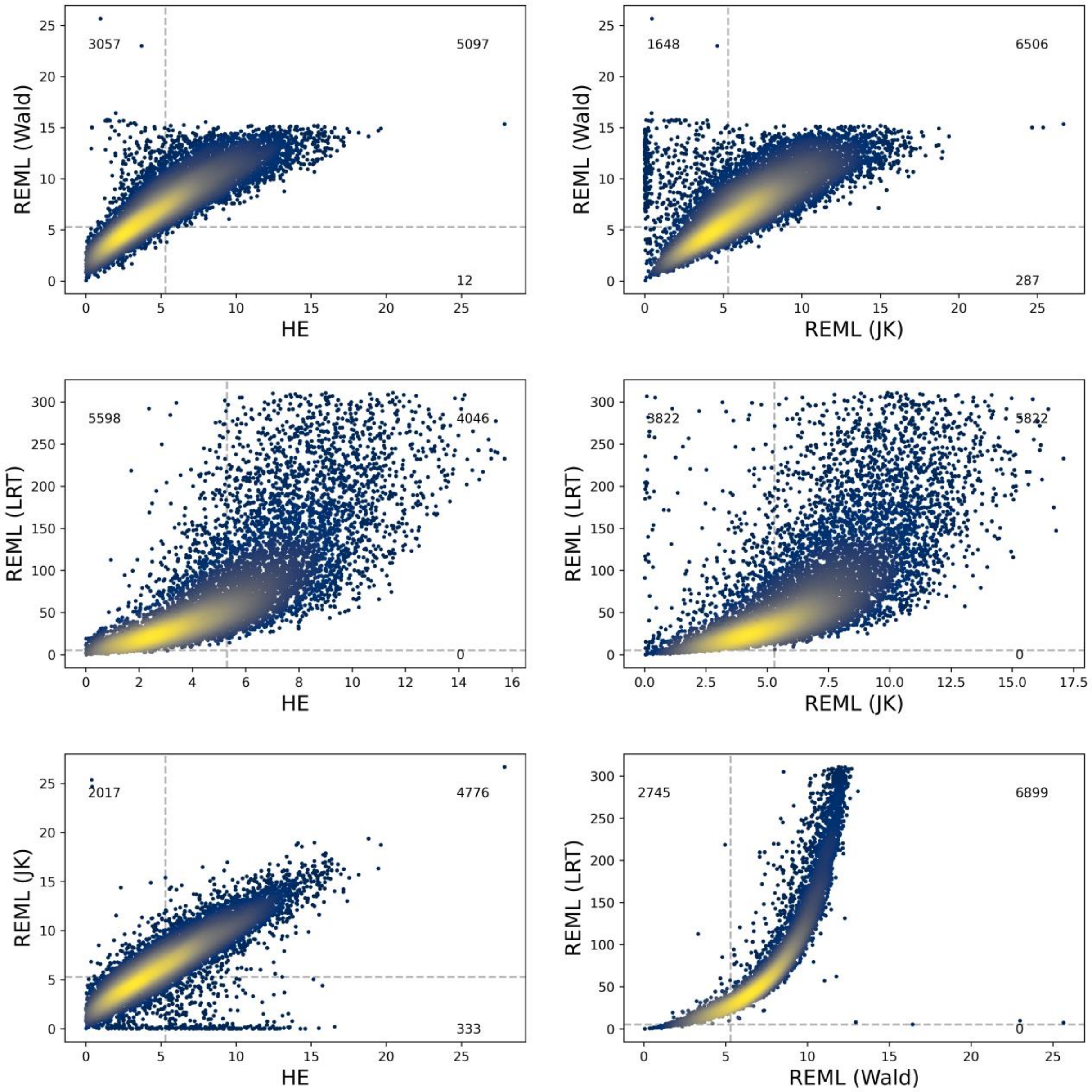

Supplementary Figure 17. Comparison of different tests for cell type-specific variance in REML and HE with CTP data. Each axis shows $-\log_{10} p(\text{variance differentiation})$. Dashed lines indicate Bonferroni-adjusted significance thresholds ($p = 0.05/number\ of\ genes$). Wald tests require the precision matrix for parameter estimates, which is based on either the inverse of the Fisher information matrix (for REML (Wald)) or jackknife (for REML (JK) and HE). Likelihood ratio tests (LRT) are also shown for REML.

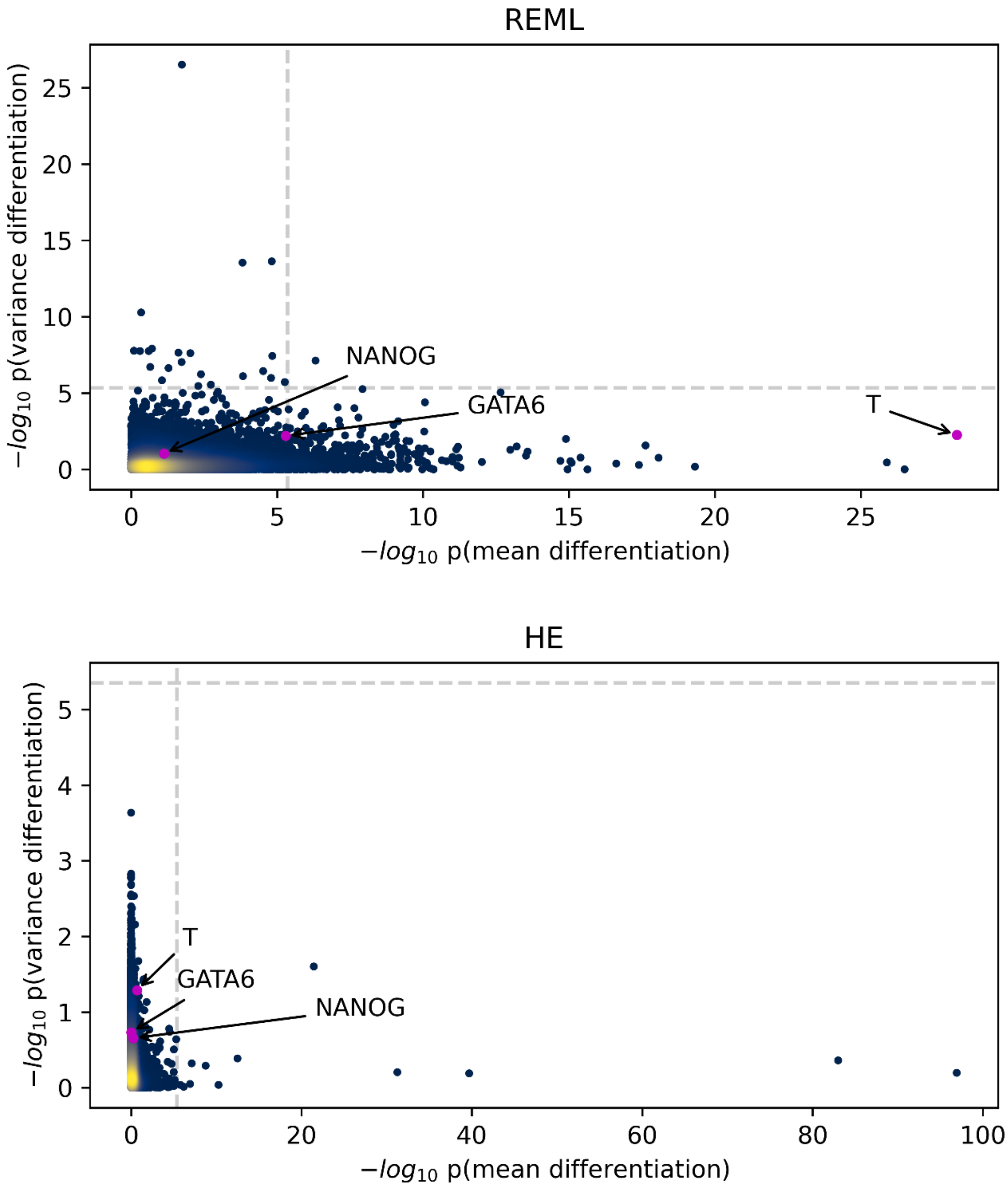

Supplementary Figure 18. Transcriptome-wide distribution of *p* values for differentiation in expression mean and variance using REML and HE with OP data. In REML, p values from Wald test were shown for mean differentiation, p values from likelihood ratio test (LRT) were shown for variance differentiation. In HE, p values for mean differentiation and variance differentiation were both from the Wald test. Each dot represents a gene. Dots are colored by the density of genes in the area, with yellow indicating denser distribution. Dashed lines indicate the significance threshold after Bonferroni correction. The three purple dots indicate the three maker genes used in *Cuomo et al*. to indicate each stem cell differentiation stage, spanning iPSC (*NANOG*), mesendoderm (*T*), and definitive endoderm (*GATA6*).

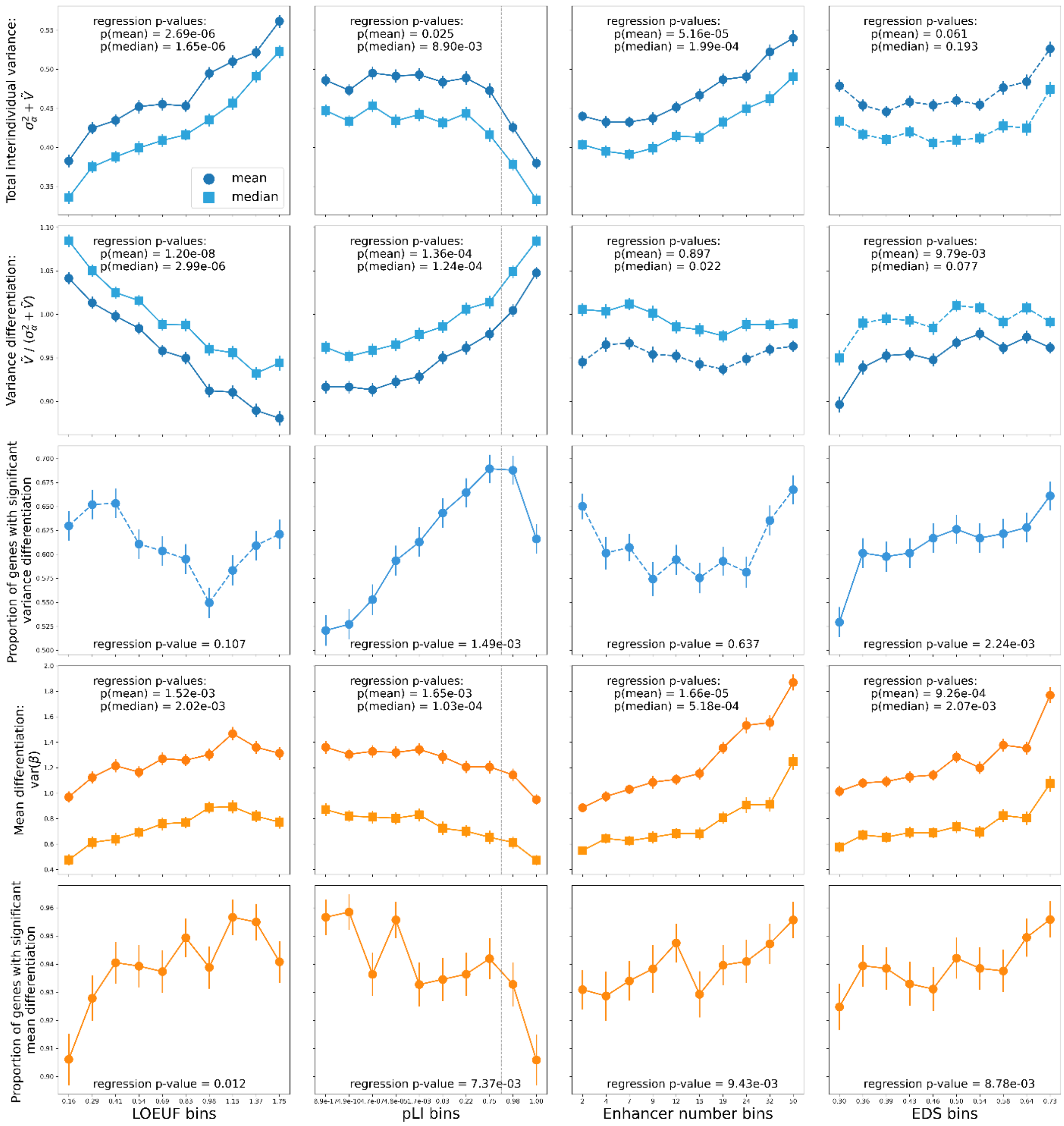

Supplementary Figure 19. Gene features associated with CTMM's interindividual variance test and ordinary mean differential expression across cell types. Each column shows one of four gene features divided into deciles across 10,891 genes, with x-axis ticks showing the feature's median value within each decile. LOEUF and pLI measure a gene's susceptibility to loss-of-function mutations, where lower LOEUF and higher pLI correspond to higher intolerance. Dashed lines in pLI mark pLI = 0.9, a common cut-off for highly constrained genes (pLI > 0.9). Enhancer number and EDS measure the enhancer structure nearby a gene, where larger enhancer numbers and larger EDS indicate larger enhancer domains. Rows show five gene expression properties from CTMM corresponding to interindividual variance (rows 1-3) and mean expression (rows 4-5) across cell types in the iPSC data. (1) The total

interindividual variance combines cell type-shared and -specific variance: $\sigma_\alpha^2 + \tilde{V}$, where $\tilde{V} = \frac{1}{C}\sum_{c=1}^{C} \mathbf{V}_{cc}$ is the average cell type-specific variance. (2) The proportion of interindividual variance that is cell type-specific, defined by $\frac{\tilde{V}}{\sigma_\alpha^2+\tilde{V}}$. (3) The proportion of genes with significant cell type-specific interindividual variance. (4) The variance of cell type-specific mean expression ($var(\beta)$). (5) The proportion of genes with significant mean differences across cell types. Displayed regression p values correspond to two-sided t-test for a simple meta-regression of each decile's mean or median against the decile index. Solid lines indicate significant meta-regressions at $p < 0.05$, while dashed lines indicate $p > 0.05$.

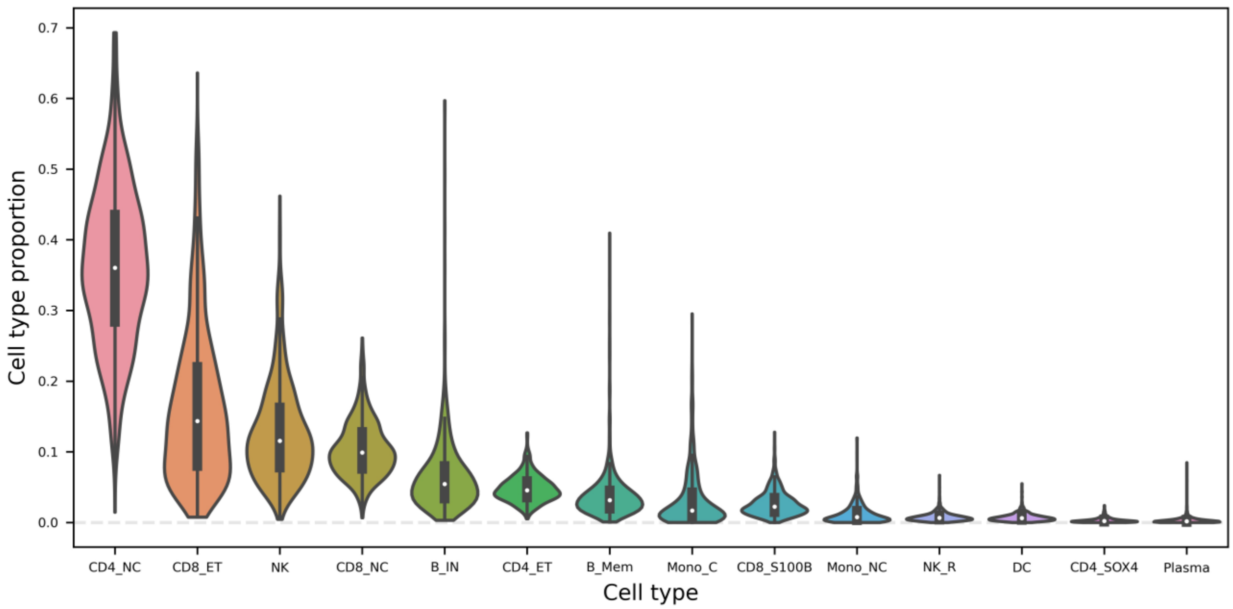

Supplementary Figure 20. Distribution of cell type proportions across 981 individuals in OneK1K. Interior box plots show the median and the first and third quartiles. The whiskers extend to values within 1.5 times the interquartile range from the first and third quartiles.

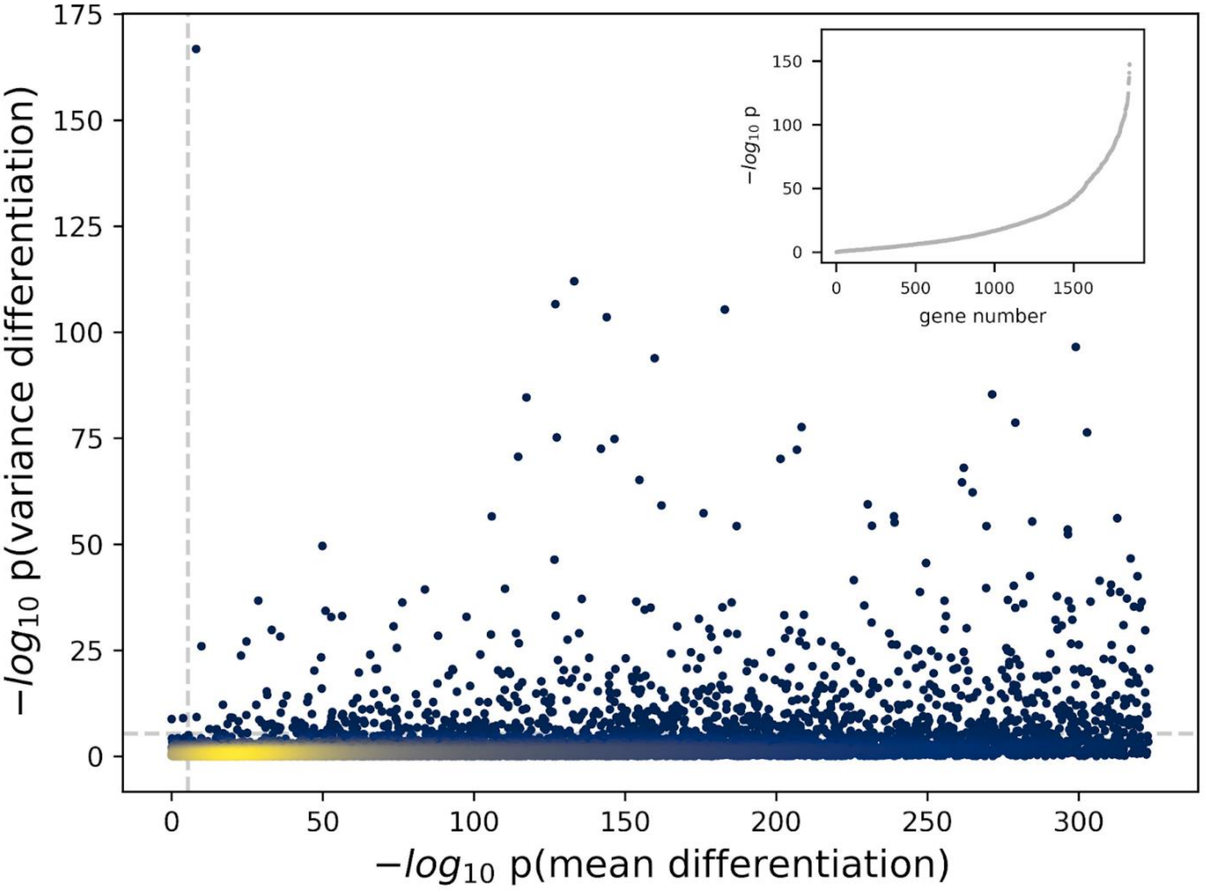

Supplementary Figure 21. Transcriptome-wide distribution of CTMM *p* values in OneK1K using HE. CTMM's test for cell type-specific interindividual variation is on the y-axis; its test for mean differential expression across cell types is on the x-axis. Each dot represents a gene, and colors reflect the density of genes in the area, with yellow indicating higher density. Dashed lines indicate the Bonferroni significance thresholds. Top right insert: the distribution of p-values from CTMM's interindividual variance test is shown for genes where the p-value for mean differential expression is essentially 0.

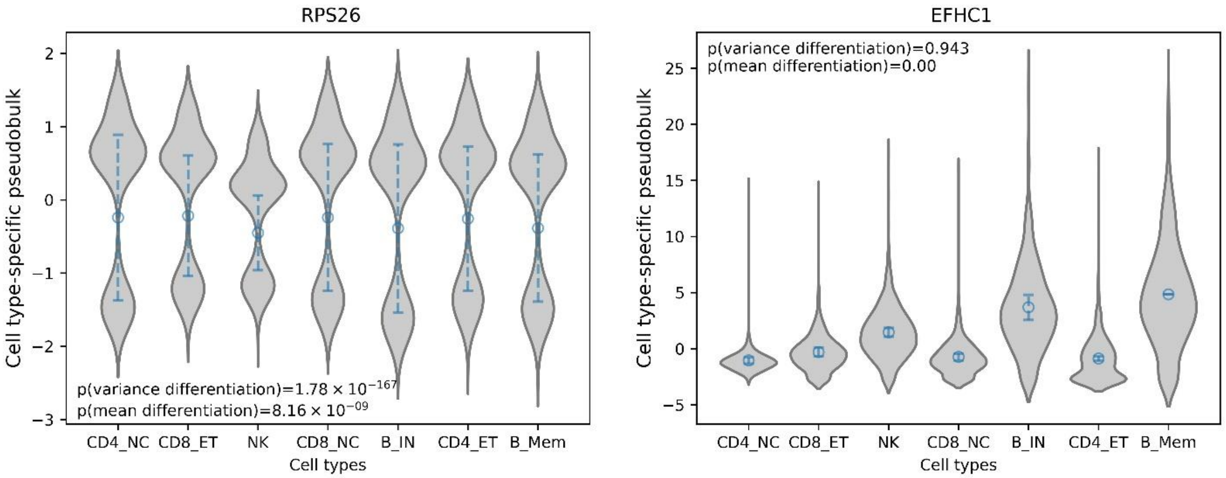

Supplementary Figure 22. Cell type-specific pseudobulk distributions across individuals for *RPS26* and *EFHC1* in OneK1K. (A) *RPS26* exhibited the strongest signal of variance differentiation among all genes, and (B) *EFHC1* exhibited the largest *p*-value of variance differentiation among genes with very strong mean differentiation (*p* = 0). P-values for testing mean differentiation and variance differentiation were calculated using the jackknife-based Wald test. The violins represent the distribution of cell type-specific pseudobulk across 928 individuals after standardizing overall pseudobulk to mean 0 and variance 1; circles indicate the estimated cell type-specific mean expression; and the dashed lines indicate two times the variance in each cell type (the sum of the homogeneous variance shared across cell types, σ_α^2, and the cell type-specific variance for that cell type, $\mathbf{V}_{CT}$).

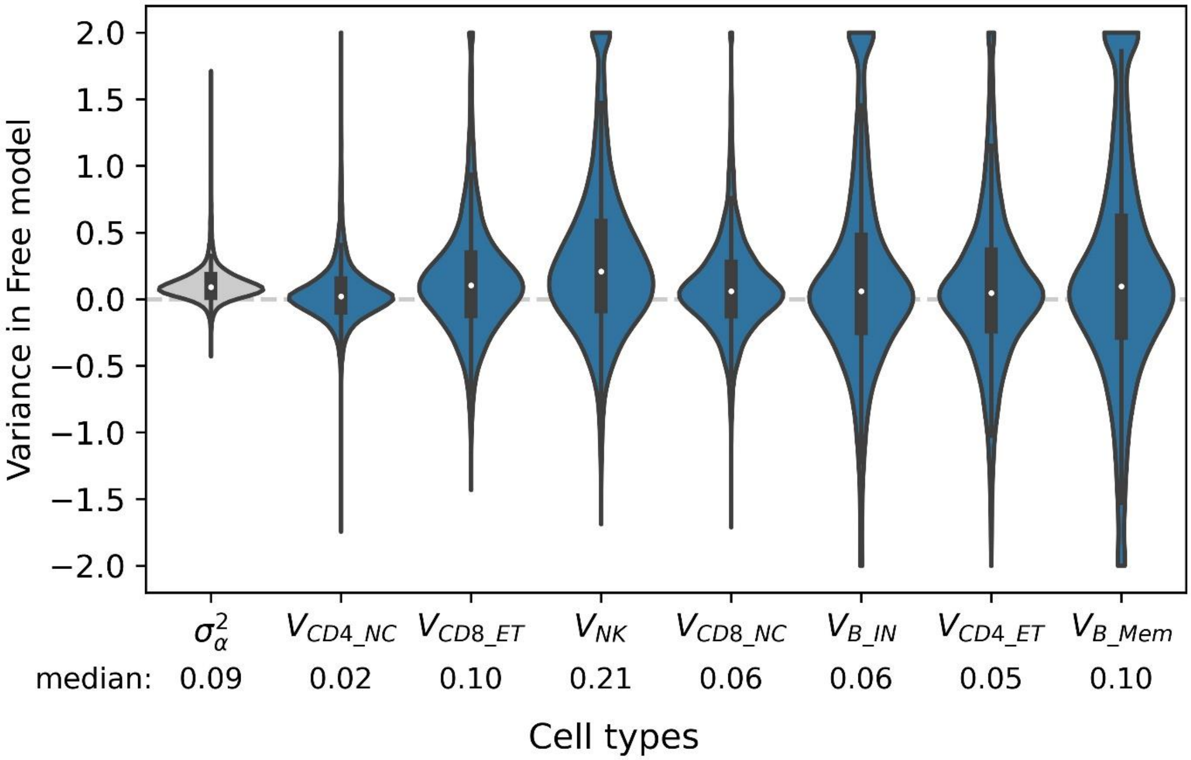

Supplementary Figure 23. Transcriptome-wide distribution of CTMM's estimates for cell type-specific interindividual variance in OneK1K. The homogeneous variance shared across cell types (σ_α^2) and the cell type-specific variance ($\mathbf{V}_{CT}$) are estimated across 11,526 genes from the Free model using HE. Violins were truncated to (-2, 2) for visibility. Cell types are ordered by cell type proportion. Interior box plots show the median and the first and third quartiles. The whiskers extend to values within 1.5 times the interquartile range from the first and third quartiles.

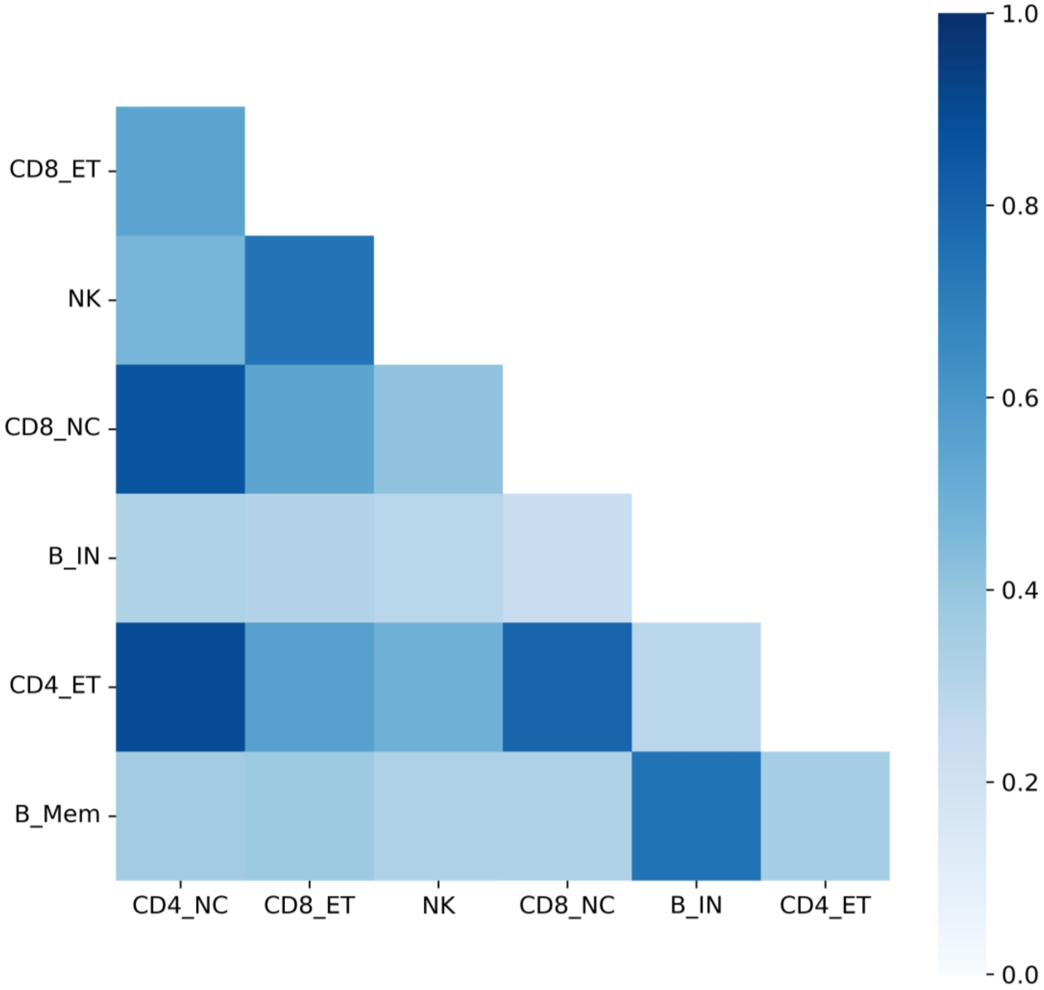

Supplementary Figure 24. Correlation of cell type-specific interindividual variation across the transcriptome in OneK1K. Each unit of the heatmap shows the median correlation across the transcriptome. 2,554 genes with positive cell type-specific variances were included. Cell types are ordered by average cell type proportions.

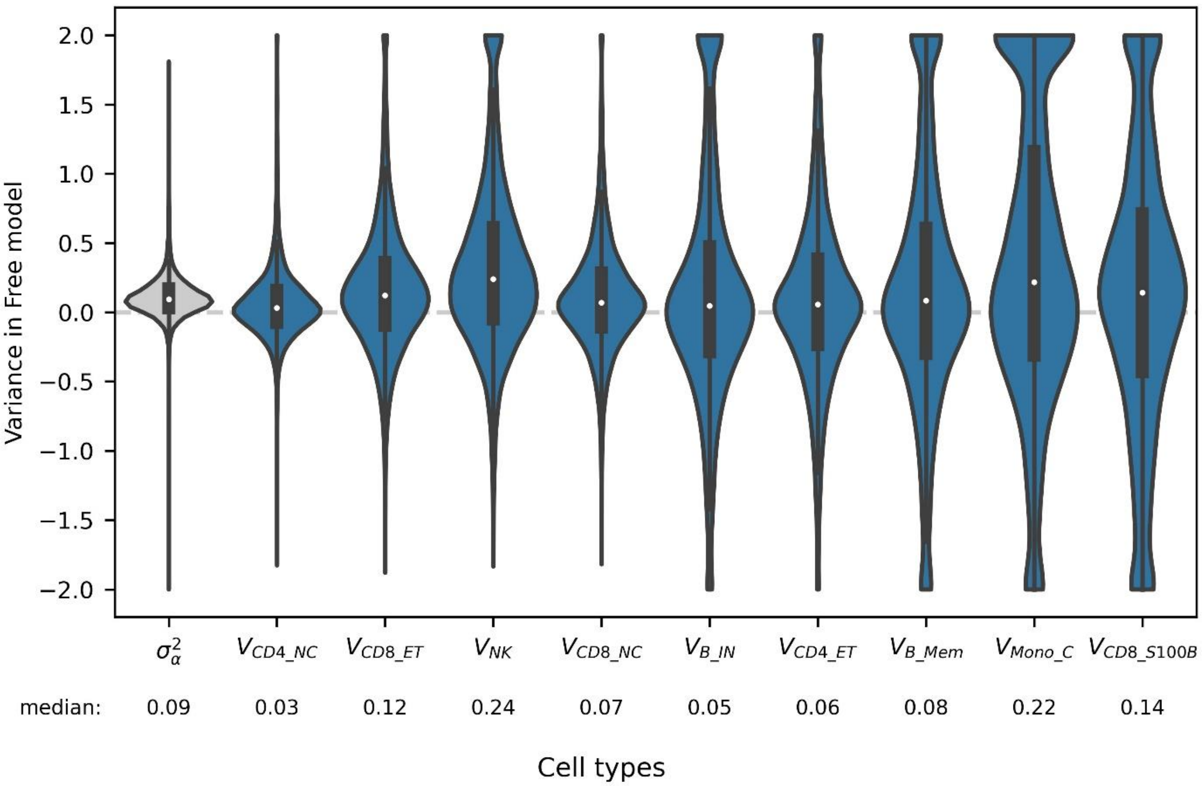

Supplementary Figure 25. Transcriptome-wide distribution of CTMM's estimates for cell type-specific interindividual variance in OneK1K including rare cell types. The homogeneous variance shared across cell types (σ_α^2) and the cell type-specific variance ($\mathbf{V}_{CT}$) are estimated across 10,768 genes from the Free model using HE. Violins were truncated to (-2, 2) for visibility. Cell types are ordered by cell type proportion. Interior box plots show the median and the first and third quartiles. The whiskers extend to values within 1.5 times the interquartile range from the first and third quartiles.

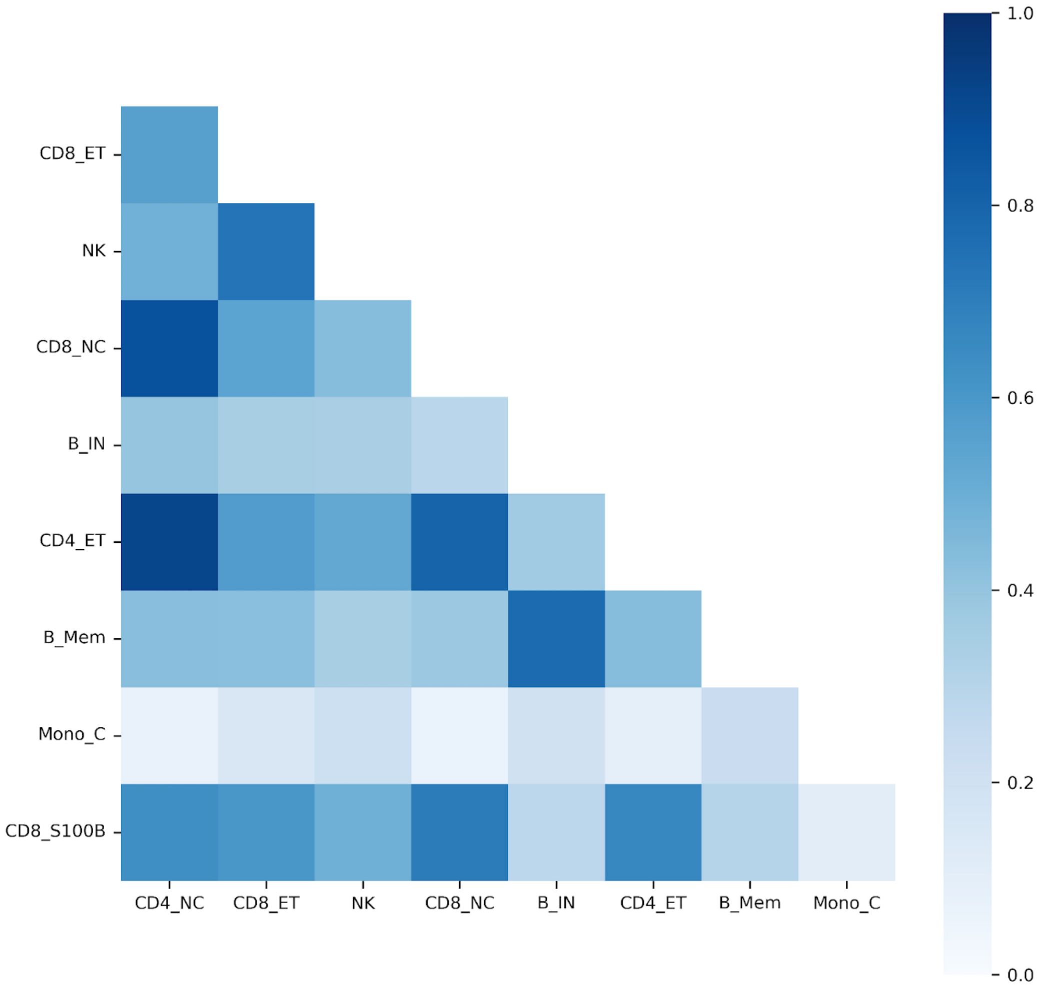

Supplementary Figure 26. Correlation of cell type-specific interindividual variation across the transcriptome in OneK1K including rarer cell types. Each unit of the heatmap shows the median correlation across the transcriptome. 1,494 genes with positive cell type-specific variances were included. Cell types are ordered by average cell type proportions.

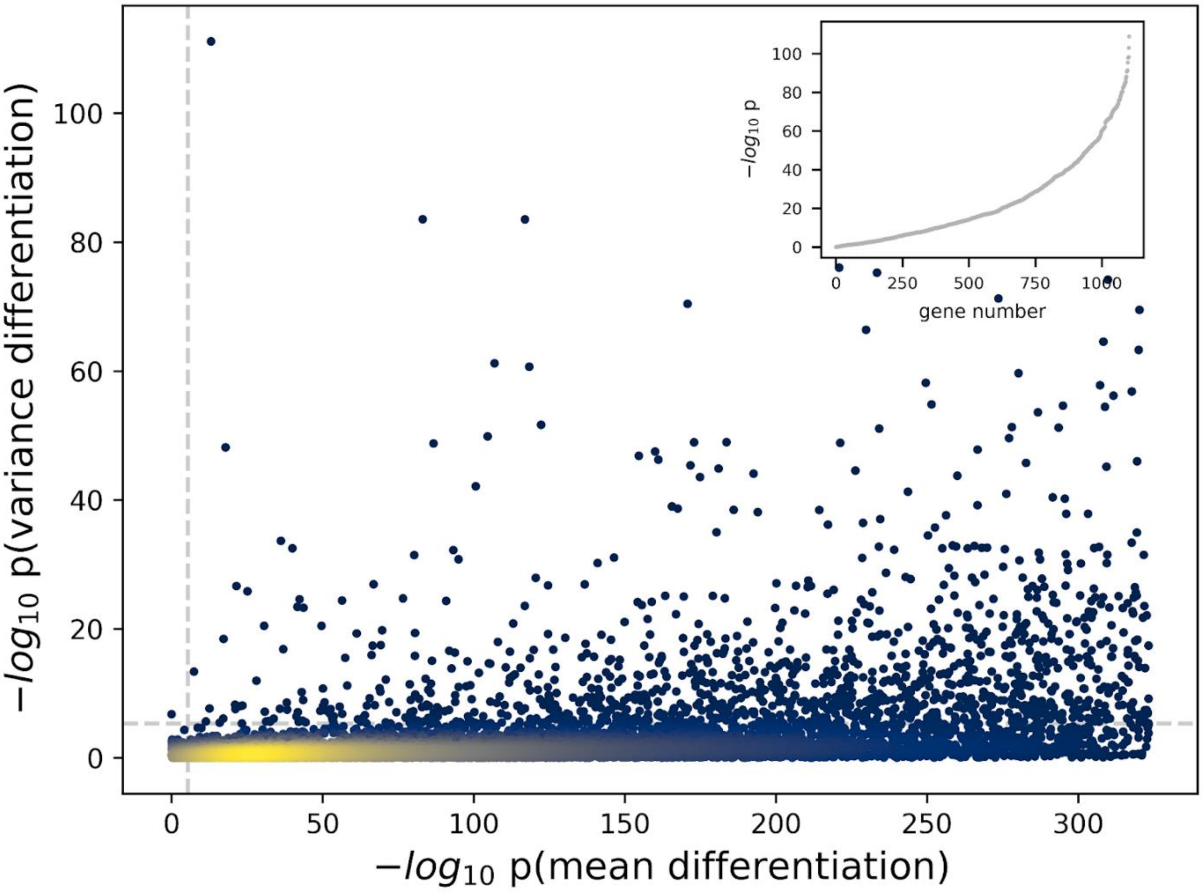

Supplementary Figure 27. Transcriptome-wide distribution of CTMM *p* values in OneK1K including rarer cell types using HE. CTMM's test for cell type-specific interindividual variation is on the y-axis; its test for mean differential expression across cell types is on the x-axis. Each dot represents a gene, and colors reflect the density of genes in the area, with yellow indicating higher density. Dashed lines indicate the Bonferroni significance thresholds. Top right insert: the distribution of *p*-values from CTMM's interindividual variance test is shown for genes where the *p*-value for mean differential expression is essentially 0.

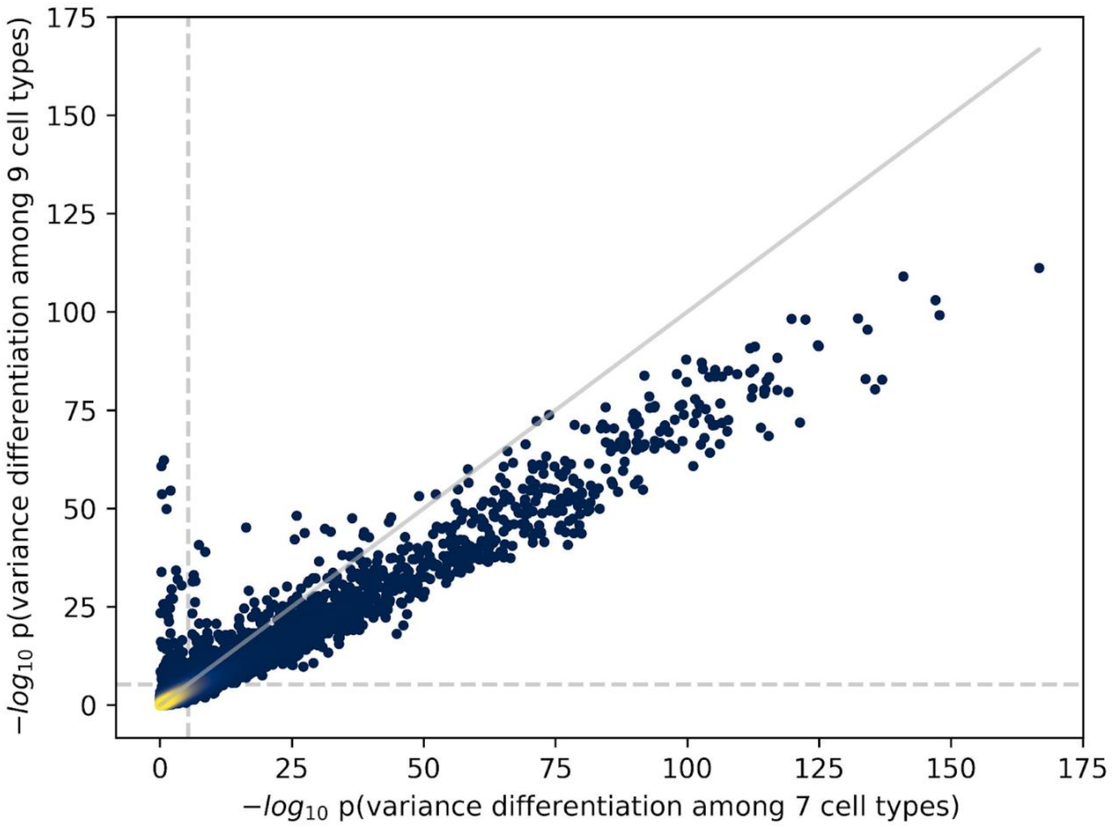

Supplementary Figure 28. Comparison of CTMM *p* values for cell type-specific interindividual variation with and without adding two rare cell types. Each dot represents a gene. Dots are colored by the density of genes in the area, with yellow indicating higher density. Dashed lines indicate the Bonferroni significance threshold. Adding rare cell types generally hurts power, but it adds significant power for many genes where interindividual variation is primarily concentrated in the rare cell types.

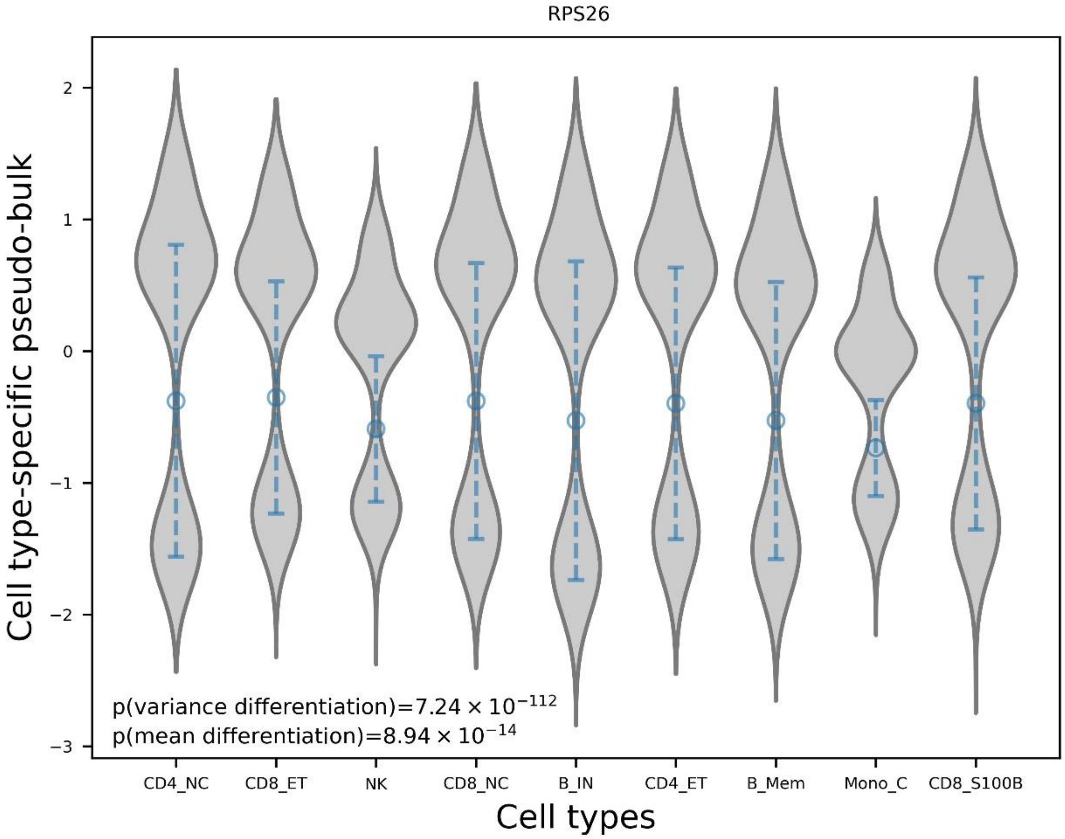

Supplementary Figure 29. Cell type-specific pseudobulk distributions across individuals for *RPS26* in OneK1K including rarer cell types. The violins represent the distribution of cell type-specific pseudobulk across 597 individuals after standardizing overall pseudobulk to mean 0 and variance 1; circles indicate the estimated cell type-specific mean expression; and the dashed lines indicate two times the variance in each cell type (the sum of the homogeneous variance shared across cell types, σ_α^2, and the cell type-specific variance for that cell type, $\mathbf{V}_{CT}$). P-values for testing mean differentiation and variance differentiation were calculated using the jackknife-based Wald test.

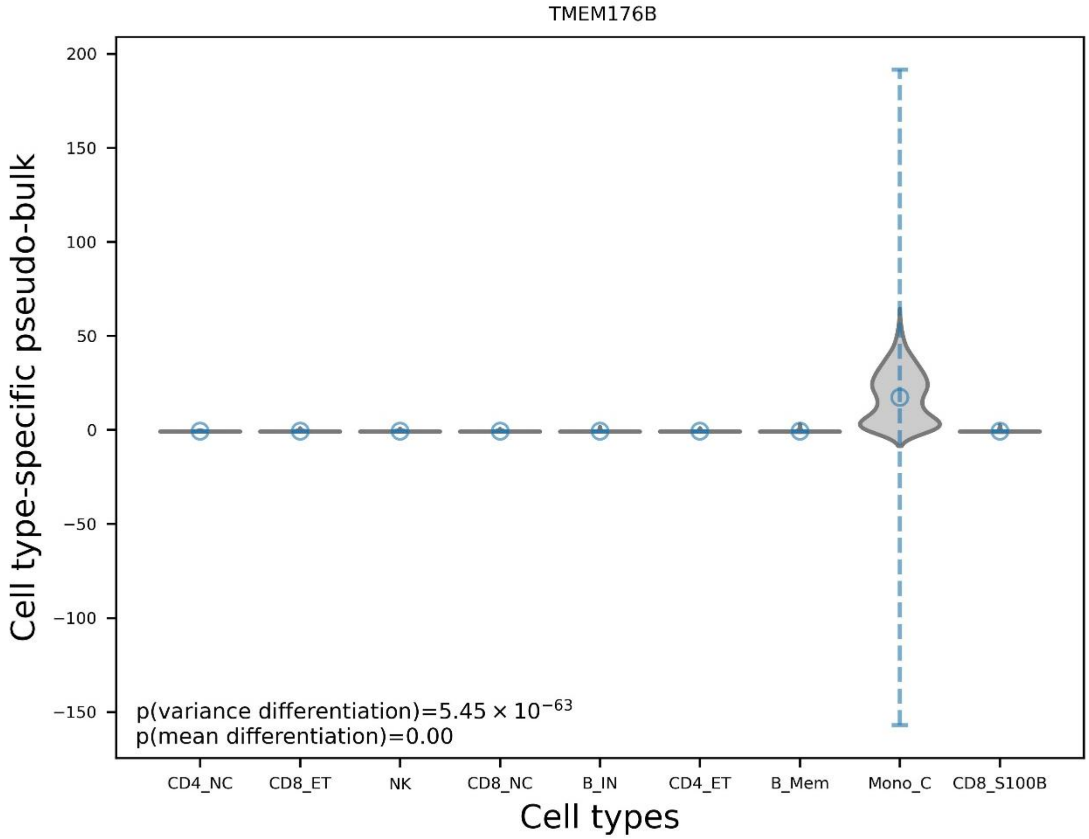

Supplementary Figure 30. Cell type-specific pseudobulk distributions across individuals for *TMEM176B* in OneK1K including rarer cell types. The violins represent the distribution of cell type-specific pseudobulk across 597 individuals after standardizing overall pseudobulk to mean 0 and variance 1; circles indicate the estimated cell type-specific mean expression; and the dashed lines indicate two times the variance in each cell type (the sum of the homogeneous variance shared across cell types, σ_α^2, and the cell type-specific variance for that cell type, $\mathbf{V}_{CT}$). P-values for testing mean differentiation and variance differentiation were calculated using the jackknife-based Wald test.

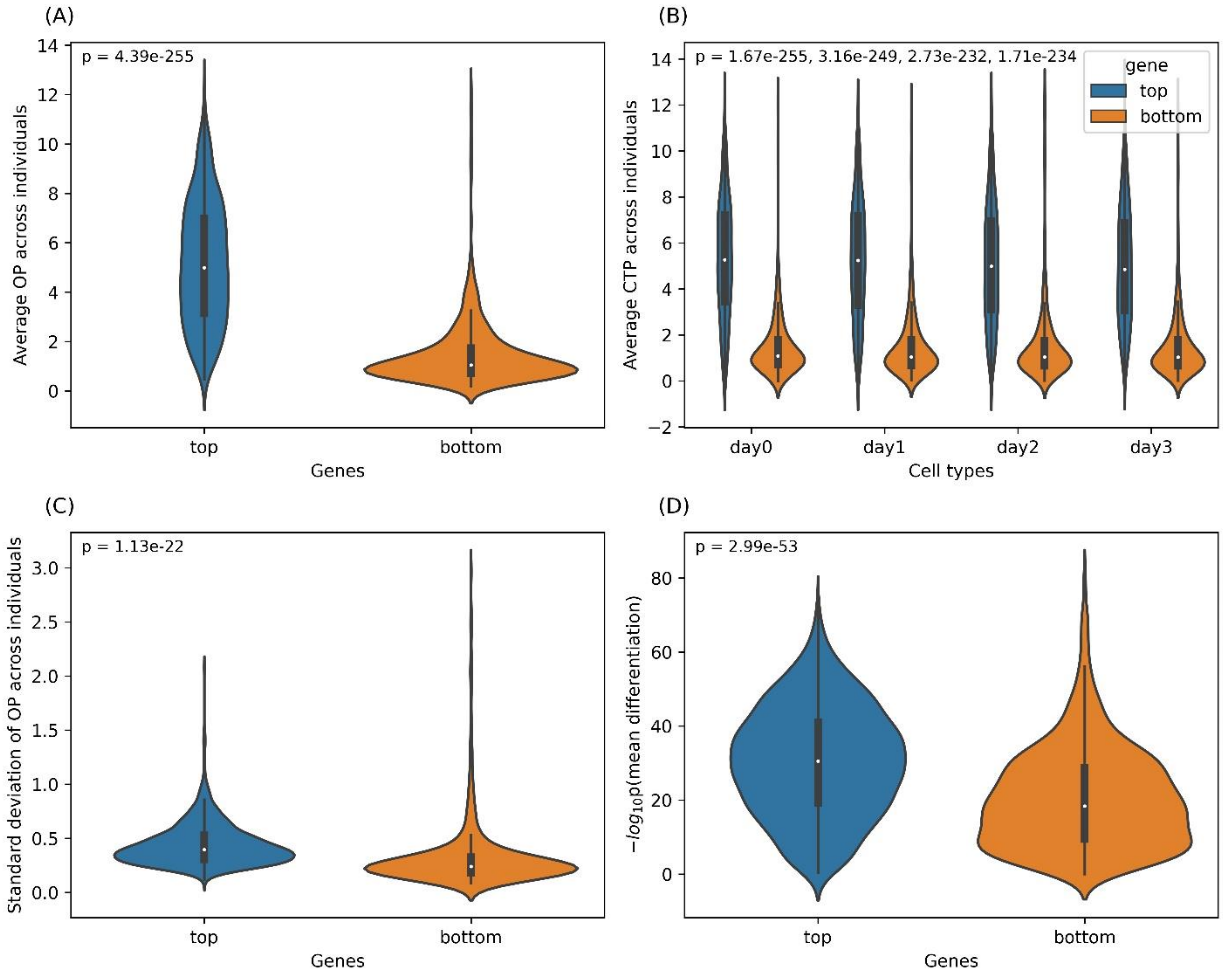

Supplementary Figure 31. Comparison between the top and bottom genes differentiated in variance in iPSCs. We selected the top (highest differentiation) 1,000 and bottom (lowest differentiation) 1,000 genes based on *p* values for CTMM's test of cell type-specific interindividual variation using REML (JK), while also requiring top genes to be significant in HE for robustness. We compared their properties in terms of each gene's (A) average overall pseudobulk expression across all individuals, (B) average cell type-specific pseudobulk expression across all individuals, (C) variance of overall pseudobulk over individuals, and (D) differentiation in mean expression across cell types. For statistical testing, *p* values for differences in (A), (B), and (C) were assessed using a two-sided *t*-test, while the difference in (D) was evaluated using a two-sided Mann-Whitney U rank test. Interior box plots show the median and the first and third quartiles. The whiskers extend to values within 1.5 times the interquartile range from the first and third quartiles.

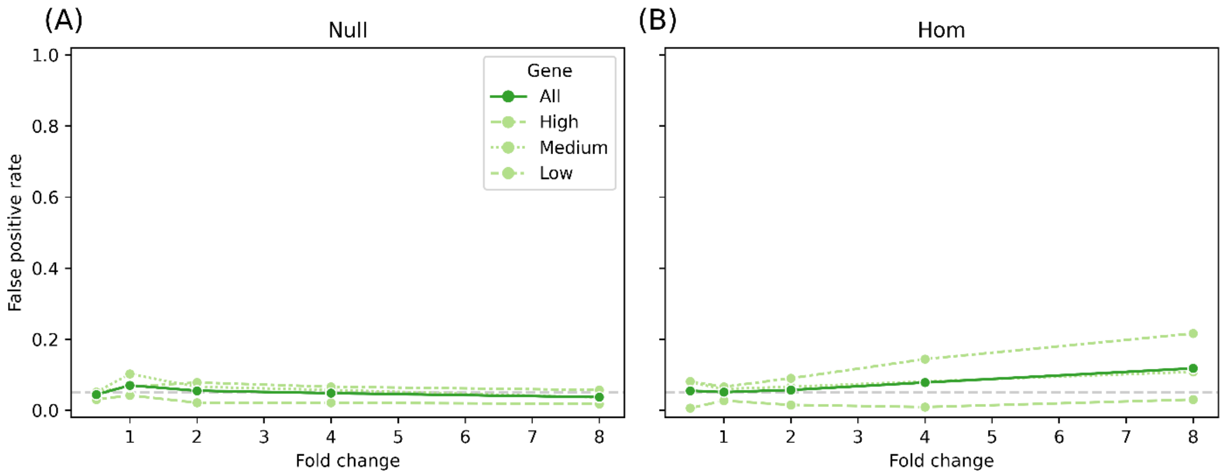

Supplementary Figure 32. False positive rate in CTMM due to mean-variance dependence across cell types. Simulations use the same framework as in Supplementary Figure 11 with known ν (left side of Supplementary Figure 11, panels A and C). (A) CTMM is unbiased regardless of the fold change in the absence of true interindividual variation (Null model). To realistically simulate from this Null model, we shuffled cells across individuals to remove all interindividual variation. (B) CTMM can be inflated for low-expressed genes under the Hom model, where interindividual variation does exist but is entirely shared across cell types. Low/Medium/High indicate tertiles of total expression across the studied genes.

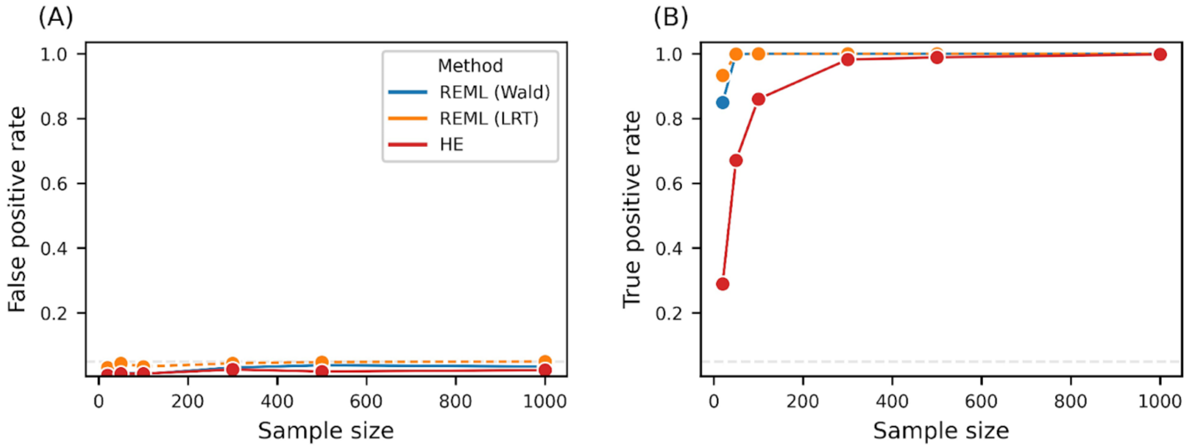

Supplementary Figure 33. Power of CTMM's test of cell type-specific variance when constraining all cell types to have the same level of cell type-specific variance. The left column shows simulations under the null Hom model, where there is no cell type-specific variance. The right column shows simulations under the alternative Free model, where each cell type has its own cell type-specific variance.

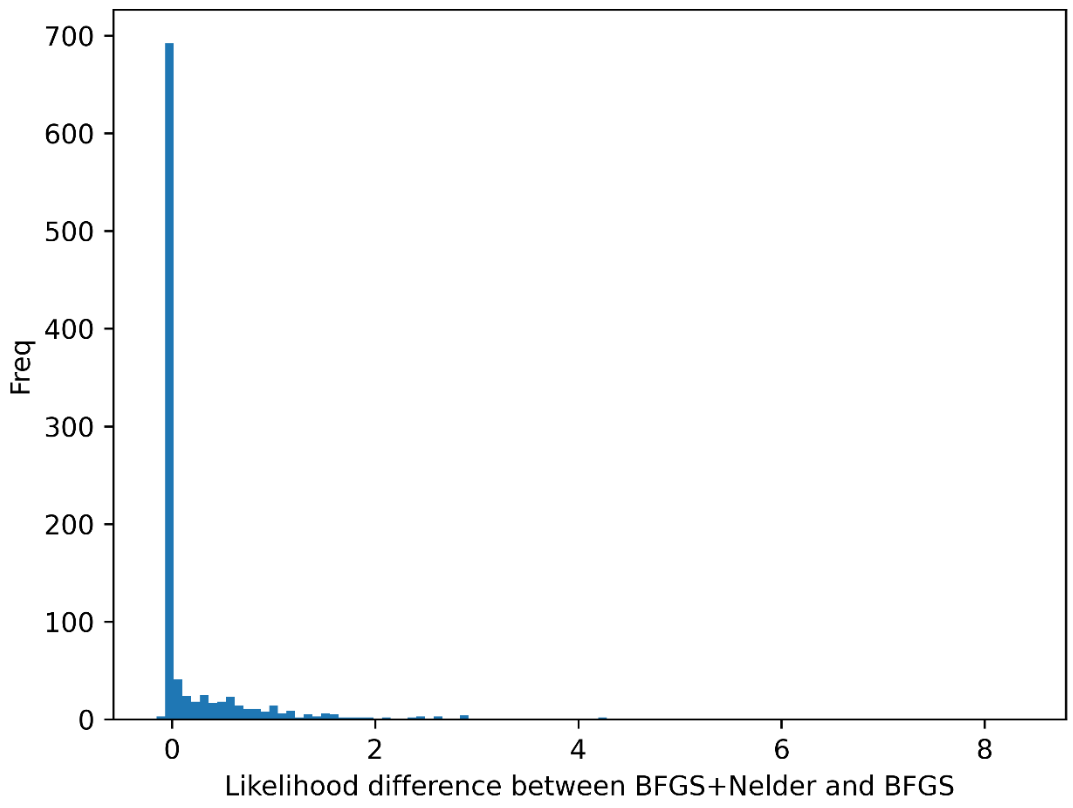

Supplementary Figure 34. Increase of inferred maximum likelihood in ML with OP after refining BFGS solution with Nelder-Mead algorithms. The same simulated OP data from the Free baseline model (with parameters shown in Supplementary Table 2) was fit by ML and optimized using BFGS alone or a combination of BFGS followed by Nelder-Mead. Both BFGS and Nelder-Mead algorithms are implemented using the R function ‘optim’.

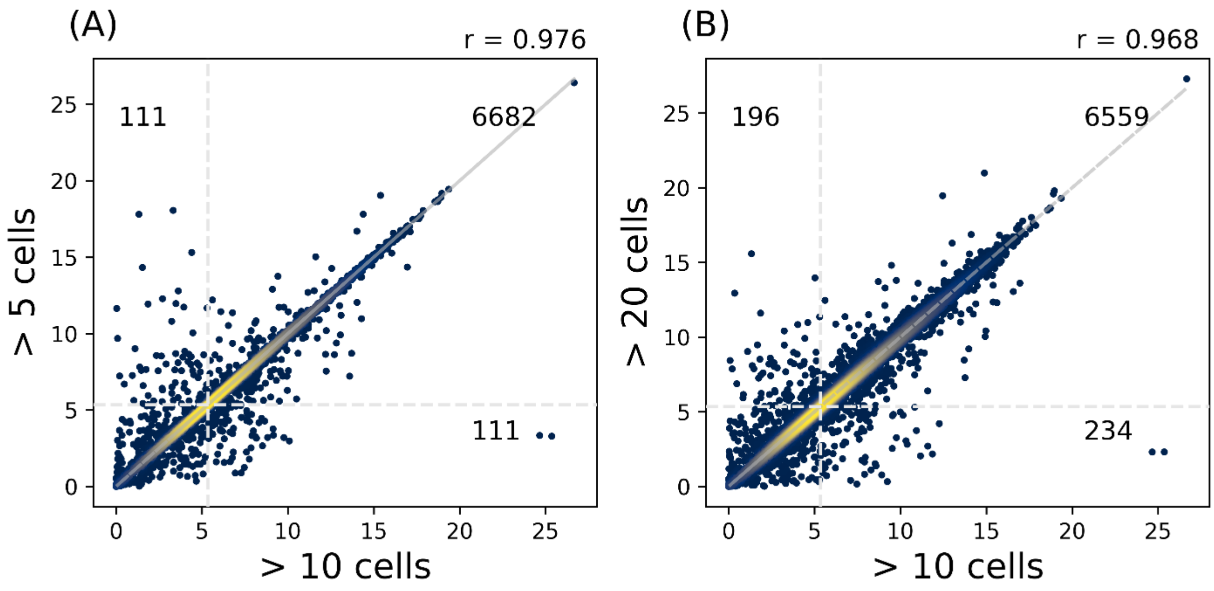

Supplementary Figure 35: CTMM's robustness to the threshold on the minimum number of cells per (individual, cell type) pair. When this number is not above the cutoff, the corresponding entry of the pseudobulk matrix is set to missing and then imputed. Plots compare CTMM's *p*-value in our primary analysis (x-axis), which requires 10 cells per (individual, cell type), to the CTMM *p*-value obtained when this is decreased to 5 (y-axis, panel A) or increased to 20 (y-axis, panel B). The numbers indicate the number of genes in each quadrant, which are defined by vertical and horizontal lines indicating Bonferroni-significance.

Supplementary References

1. Cuomo, A.S.E. *et al.* Single-cell RNA-sequencing of differentiating iPS cells reveals dynamic genetic effects on gene expression. *Nat Commun* **11**, 1-14 (2020).
2. Yazar, S. *et al.* Single-cell eQTL mapping identifies cell type-specific genetic control of autoimmune disease. *Science (1979)* **376**, (2022).
3. Tung, P. Y. *et al.* Batch effects and the effective design of single-cell gene expression studies. *Scientific Reports 2017 7:1* **7**, 1–15 (2017).
4. Chung, Y., Rabe-Hesketh, S., Dorie, V., Gelman, A. & Liu, J. A nondegenerate penalized likelihood estimator for variance parameters in multilevel models. *Psychometrika* **78**, 685–709 (2013).
5. Martinez-Jimenez, C. P. *et al.* Aging increases cell-to-cell transcriptional variability upon immune stimulation. *Science (1979)* **355**, 1433–1436 (2017).
6. Vallejos, C. A., Richardson, S. & Marioni, J. C. Beyond comparisons of means: Understanding changes in gene expression at the single-cell level. *Genome Biol* **17**, (2016).
7. Crowell, H. L. *et al.* muscat detects subpopulation-specific state transitions from multi-sample multi-condition single-cell transcriptomics data. *Nature Communications 2020 11:1* **11**, 1–12 (2020).
8. Bates, D., Mächler, M., Bolker, B. M. & Walker, S. C. Fitting Linear Mixed-Effects Models Using lme4. *J Stat Softw* **67**, 1–48 (2015).
9. Hoffman, G. E. & Schadt, E. E. variancePartition: Interpreting drivers of variation in complex gene expression studies. *BMC Bioinformatics* **17**, 1–13 (2016).
10. Cuomo, A. S. E. *et al.* Single-cell RNA-sequencing of differentiating iPS cells reveals dynamic genetic effects on gene expression. *Nat Commun* **11**, 1–14 (2020).
11. Casale, F. P., Rakitsch, B., Lippert, C. & Stegle, O. Efficient set tests for the genetic analysis of correlated traits. *Nature Methods 2015 12:8* **12**, 755–758 (2015).
12. Cuomo, A. S. E. *et al.* CellRegMap: a statistical framework for mapping context-specific regulatory variants using scRNA-seq. *Mol Syst Biol* **18**, e10663 (2022).
