# Supplementary material for: A robust model for cell type-specific interindividual variation in single-cell RNA sequencing data
Source: Nat Commun. 2024 Jun 19;15:5229. doi: 10.1038/s41467-024-49242-9 (PMC11186839; doi:10.1038/s41467-024-49242-9)
Supplement: Supplementary file 3 — Description of Additional Supplementary Files [file 41467_2024_49242_MOESM3_ESM.pdf]

Description of Additional Supplementary Files

File name: Supplementary Data 1

Description: GO enrichment analysis on the top 100 genes with significantly differentiated variance but no mean differentiation after Bonferroni correction. P-values were calculated by hypergeometric distribution and adjusted for multiple comparisons using the BH method.

File name: Supplementary Data 2

Description: GO enrichment analysis on the top 100 genes with significantly differentiated variance in OneK1K. P-values were calculated by hypergeometric distribution and adjusted for multiple comparisons using the BH method.
