# Supplementary material for: A robust model for cell type-specific interindividual variation in single-cell RNA sequencing data
Source: Nat Commun. 2024 Jun 19;15:5229. doi: 10.1038/s41467-024-49242-9 (PMC11186839; doi:10.1038/s41467-024-49242-9)
Supplement: Supplementary file 6 — Reporting Summary [file 41467_2024_49242_MOESM6_ESM.pdf]

nature portfolio

Corresponding author(s): Andy Dahl

Last updated by author(s): Apr 18, 2024

Reporting Summary

Nature Portfolio wishes to improve the reproducibility of the work that we publish. This form provides structure for consistency and transparency in reporting. For further information on Nature Portfolio policies, see our Editorial Policies and the Editorial Policy Checklist.

Statistics

For all statistical analyses, confirm that the following items are present in the figure legend, table legend, main text, or Methods section.

n/a	Confirmed	
☐	☒	The exact sample size (*n*) for each experimental group/condition, given as a discrete number and unit of measurement
☐	☒	A statement on whether measurements were taken from distinct samples or whether the same sample was measured repeatedly
☐	☒	The statistical test(s) used AND whether they are one- or two-sided<br>*Only common tests should be described solely by name; describe more complex techniques in the Methods section.*
☐	☒	A description of all covariates tested
☐	☒	A description of any assumptions or corrections, such as tests of normality and adjustment for multiple comparisons
☐	☒	A full description of the statistical parameters including central tendency (e.g. means) or other basic estimates (e.g. regression coefficient) AND variation (e.g. standard deviation) or associated estimates of uncertainty (e.g. confidence intervals)
☐	☒	For null hypothesis testing, the test statistic (e.g. *F*, *t*, *r*) with confidence intervals, effect sizes, degrees of freedom and *P* value noted<br>*Give P values as exact values whenever suitable.*
☒	☐	For Bayesian analysis, information on the choice of priors and Markov chain Monte Carlo settings
☒	☐	For hierarchical and complex designs, identification of the appropriate level for tests and full reporting of outcomes
☒	☐	Estimates of effect sizes (e.g. Cohen's *d*, Pearson's *r*), indicating how they were calculated

Our web collection on statistics for biologists contains articles on many of the points above.

Software and code

Policy information about availability of computer code

Data collection	No software was used.
Data analysis	Data were analyzed using Python (3.11.5), R (4.3.1), CTMM Python package (https://github.com/Minhui-Chen/CTMM), and countsimQC.

For manuscripts utilizing custom algorithms or software that are central to the research but not yet described in published literature, software must be made available to editors and reviewers. We strongly encourage code deposition in a community repository (e.g. GitHub). See the Nature Portfolio guidelines for submitting code & software for further information.

Data

Policy information about availability of data

All manuscripts must include a data availability statement. This statement should provide the following information, where applicable:

- Accession codes, unique identifiers, or web links for publicly available datasets
- A description of any restrictions on data availability
- For clinical datasets or third party data, please ensure that the statement adheres to our policy

Processed single cell count data from iPSCs are available from Zenodo: https://zenodo.org/record/3625024#.Xil-0y2cZ0s. OneK1K single-cell gene expression data are available via Gene Expression Omnibus (GSE196830 [https://www.ncbi.nlm.nih.gov/geo/query/acc.cgi?acc=GSE196830]).

Research involving human participants, their data, or biological material

Policy information about studies with human participants or human data. See also policy information about sex, gender (identity/presentation), and sexual orientation and race, ethnicity and racism.

Reporting on sex and gender	Our data are roughly balanced between males and females. We did not perform stratified analyses based on sex and/or gender.
Reporting on race, ethnicity, or other socially relevant groupings	Human iPSC lines were derived from 125 unrelated British donors from the Human Induced Pluripotent Stem Cell initiative (HipSci). OneK1K participants mostly self-reported as Northern European ancestry. We didn't correct for race or ethnicity in our analyses.
Population characteristics	Eleven iPSC lines were derived from neonatal diabetes patients, while the remaining lines were derived from healthy donors. The OneK1K study consist of ~1,000 participants ranged from 19 to 97, with 73% of participants being older than 60 years old. All participants were without active immune disease except one with giant cell arthritis which was removed from analysis.
Recruitment	Human iPSC lines were derived from contented research volunteers recruited from the NIHR Cambridge BioResource. OneK1K samples were ascertained from patients or their relatives attending the Royal Hobart Hospital, Hobart Eye Surgeons as well as individuals residing in the retirement villages within Hobart, Australia.
Ethics oversight	Recruitment of volunteers to derive iPSC lines were approved under ethics for iPS cell derivation (REC 09//H0304/77, V2 04/01/2013, and revised consent REC 09/H0304/77, V3 15/03/2013). OneK1K study was approved by the Tasmania Health and Medical Human Research Ethics Committee (H0012902).

Note that full information on the approval of the study protocol must also be provided in the manuscript.

Field-specific reporting

Please select the one below that is the best fit for your research. If you are not sure, read the appropriate sections before making your selection.

☒ Life sciences ☐ Behavioural & social sciences ☐ Ecological, evolutionary & environmental sciences

For a reference copy of the document with all sections, see nature.com/documents/nr-reporting-summary-flat.pdf

Life sciences study design

All studies must disclose on these points even when the disclosure is negative.

Sample size	We excluded individuals with a limited number of cells in order to improve the Gaussian approximation of pseudobulk gene expression. After this exclusion, we retained 94 individuals for the iPSC study and 928 for the OneK1K study. Our simulations demonstrate that these sample sizes provide ample statistical power.
Data exclusions	In the iPSC study, we excluded individuals with fewer than 100 cells. In the OneK1K study, we excluded cell types that had 10 or fewer cells in more than 10% of the cohort's individuals. Subsequently, we excluded individuals that had cell types with 10 or fewer cells. These exclusions were implemented to better satisfy the Gaussian approximation of pseudobulk gene expression. As there were no established exclusion criteria, we applied these exclusion criteria as a general guidance.
Replication	We validated our findings by aligning them with established literature, demonstrating their consistency with previous research. We compared our variance-differentiated genes with previously identified markers for cell differentiation and pluripotency, as well as eGenes documented in the previous OneK1K cohort study. We also replicated the relationship between cell types observed in the iPSC and OneK1K studies.
Randomization	In the iPSC study, cell lines were allocated to experimental batches at random, with 4-6 cell lines co-cultured and grown as a mixed population for a total of 28 experiments, in 12 well plates. In the OneK1K study, live cells were randomly pooled with 12 to 14 participant samples per pool.
Blinding	Blinding is not relevant to our study. Additionally, we do not have access to any identifiable individual-level data.

Reporting for specific materials, systems and methods

We require information from authors about some types of materials, experimental systems and methods used in many studies. Here, indicate whether each material, system or method listed is relevant to your study. If you are not sure if a list item applies to your research, read the appropriate section before selecting a response.

Materials & experimental systems

n/a	Involved in the study
☒	☐ Antibodies
☒	☐ Eukaryotic cell lines
☒	☐ Palaeontology and archaeology
☒	☐ Animals and other organisms
☒	☐ Clinical data
☒	☐ Dual use research of concern
☒	☐ Plants

Methods

n/a	Involved in the study
☒	☐ ChIP-seq
☒	☐ Flow cytometry
☒	☐ MRI-based neuroimaging

Plants

Seed stocks	*Report on the source of all seed stocks or other plant material used. If applicable, state the seed stock centre and catalogue number. If plant specimens were collected from the field, describe the collection location, date and sampling procedures.*
Novel plant genotypes	*Describe the methods by which all novel plant genotypes were produced. This includes those generated by transgenic approaches, gene editing, chemical/radiation-based mutagenesis and hybridization. For transgenic lines, describe the transformation method, the number of independent lines analyzed and the generation upon which experiments were performed. For gene-edited lines, describe the editor used, the endogenous sequence targeted for editing, the targeting guide RNA sequence (if applicable) and how the editor was applied.*
Authentication	*Describe any authentication procedures for each seed stock used or novel genotype generated. Describe any experiments used to assess the effect of a mutation and, where applicable, how potential secondary effects (e.g. second site T-DNA insertions, mosiacism, off-target gene editing) were examined.*
